# Health anxiety amplifies fearful responses to illness-related imagery

**DOI:** 10.1038/s41598-024-54985-y

**Published:** 2024-02-22

**Authors:** Christoph Benke, Laura-Marie Wallenfels, Gaby M. Bleichhardt, Christiane A. Melzig

**Affiliations:** 1 Department of Psychology, Clinical Psychology and Psychotherapy, Philipps University of Marburg, Gutenbergstraße 18, 35032 Marburg, Germany; 2 Center for Mind, Brain and Behavior (CMBB), University of Marburg and Justus Liebig University Giessen, Giessen, Germany

**Keywords:** Illness anxiety, Mental images, Anxiety, Body symptoms, Psychology, Anxiety

## Abstract

Severe health anxiety (HA) is characterized by excessive worry and anxiety about one's health, often accompanied by distressing intrusive imagery of signs of a serious illness or potentially receiving bad news about having a life-threatening disease. However, the emotional responses to these illness-related mental images in relation to HA have not been fully elucidated. Emotional responses to mental imagery of 142 participants were assessed in a well-controlled script-driven imagery task, systematically comparing emotional responses to illness-related imagery with neutral and standard fear imagery. The results revealed that participants reported higher anxiety, aversion, emotional arousal, and a stronger avoidance tendency during imagery of fear and illness-related scenes compared to neutral scenes. Importantly, the emotional modulation varied by the level of HA, indicating that individuals with higher HA experienced stronger emotional responses to illness-related imagery. This association between HA and fearful imagery could not be better accounted for by other psychological factors such as trait anxiety, anxiety sensitivity, somatic symptom severity, or symptoms of depression and anxiety. Fearful responding to standard threat material was not associated with HA. The present findings highlight the importance of considering fear responding to mental imagery in understanding and addressing HA.

## Introduction

Anxiety and worries related to one’s health are common^1,2^, especially in times of a pandemic where individuals are faced with a potential threat of contracting a disease^3,4^. However, individuals with severe health anxiety (HA) suffer from *excessive and persistent* worry and anxiety about one’s health, having or getting a severe disease, concomitant with avoidance behavior, body checking or reassurance behavior^1,5^. Health anxiety is a dimensional construct that shares characteristics with two specific DSM-5 diagnoses, i.e. illness anxiety disorder and somatic symptom disorder^6^, but may also manifest in various other disorders, such as panic disorder, where health concerns are part of a broader symptomatology^7^. It has been demonstrated that most of the individuals with severe HA reported distressing intrusive imagery centered around situations of suffering from a serious illness or getting bad news about having a life-threatening disease^8^. Ample evidence in anxiety disorders indicates that such mental images involving disorder-relevant situations (e.g., being in a crowded area, or experiencing a panic attack) may activate defensive brain circuits that entail the elicitation of fear, arousal, and avoidance or safety behavior^9–12^, thus contributing to the maintenance of anxiety psychopathology^13,14^. Although it has previously been presumed that mental images may play a role in HA^15^, emotional responses to illness-related mental images in relation to HA have not yet been elucidated. Examining the link between HA and responses to illness-related mental images would, indeed, allow to advance our understanding of psychopathologically relevant processes (i.e., fearful mental imagery of contracting a life-threatening disease) and inform prevention and intervention in individuals with severe HA.

First indication for the role of illness-related mental images in HA comes from a recent study that linked HA to fearful imagery of contracting COVID-19 by systematically comparing mental imagery of COVID-19-related situations with standard fear and non-aversive (neutral) situations in an experimental design^16^. It was demonstrated that HA was associated with a more pronounced fearful response during mental imagery of COVID-19 scripts, while no associations with other relevant psychological factors (i.e., anxiety sensitivity, trait anxiety, or depressive and anxiety symptoms) were observed. Moreover, there was no association of HA with fear responses to imagery of standard fear scripts, suggesting a specific sensitivity to fearfully respond to stimuli or context information regarding a potential infection with COVID-19—at the time of assessment considered as a life-threatening disease—in individuals with higher levels of HA^16^.

Building upon this evidence, in the present study, we targeted at examining the relationship between HA and emotional responses to mental images centered around themes commonly reported by individuals with severe HA, i.e. suffering from a serious illness or getting bad news about having a life-threatening disease^8^, and thus go beyond mental images that are only relevant in time of a pandemic. To this end, we assessed HA and elicited illness-related mental images in a controlled experimental design to characterize the emotional responses to these images^10,11,16,17^. Moreover, we aimed to examine the role of fearful responding to body symptoms in HA. Individuals with severe HA typically exhibit an interpretation bias^18,19^ in that they misinterpret actually benign body symptoms as signs of potentially life-threatening diseases^20,21^, suggesting that body symptoms may trigger the brain’s fear system concomitant with maladaptive fear responses and anxious behavior. To further elucidate the potential role of fearful responding to actually innocuous body symptoms in HA, in the present study, we also included mental imagery of narrative scenes involving body symptoms such as headache or dizziness without providing further illness-related concerns or context information. Imagery responses were systematically compared to neutral scripts as a non-aversive control condition as well as to standard fear (survival threat) situations which are known to elicit defensive mobilization^9,22,23^. We assessed verbal indicators of defensive activation during imagery including hedonic valence as well as arousal, experienced anxiety, and the tendency to avoid imagery. Moreover, we tested for the specificity of the potential associations with HA, that is, whether fearful imagery cannot be better explained by variables that have previously been demonstrated to be associated with fearful responses to imagery and/or body symptoms (e.g., anxiety sensitivity, somatic symptom severity)^24–26^.

In the present study, we expected that participants would report higher anxiety, aversion, arousal, and a stronger avoidance tendency during imagery of standard fear and illness-related scripts as compared to the imagery of the neutral scripts. Moreover, we expected that higher HA would be associated with higher anxiety, aversion, arousal, and a stronger avoidance tendency during imagery of illness-related narrative scenes including those focusing on body symptoms without illness-related context information as compared to neutral scripts. Based on our previous study and studies in anxiety disorders^12,22,27^, we assumed that HA will not be associated with fearful responses to imagery of standard threat material that has no specific relevance for individuals with high HA (e.g., being attacked by a snake).

## Methods

### Participants

In the present study, 175 participants completed an online experiment that was conducted between the 9th June and 29th July 2021. Thirty-three participants (19%) were excluded from the present analyses as they reported that they were interrupted, disturbed, or distracted during the experiment. Overall, 142 participants (71.1% women) were included in the present analyses. Participants were aged 18 to 76 (*M* = 28.15, *SD* = 11.90). A further characterization of the sample is summarized in Table 1. Participants were recruited via convenience sampling methods (social media, personal contacts, emails, etc.). All participants were required to be at least 18 years old. No further inclusion or exclusion criteria were applied. All participants gave their informed consent. The study was approved by the Ethics Committee of the Department of Psychology at the University of Marburg, Germany. All methods were performed in accordance with the relevant guidelines and regulations.

**Table 1:** Sample characteristics.

	*M*	*SD*
Age	28.15	11.90
Gender (female, %)	71.1%	
anxiety sensitivity [ASI-3]	21.37	14.43
health anxiety [IAS]	22.35	12.96
anxiety symptoms [GAD-2]	1.76	1.61
somatic symptom severity [PHQ-15]	7.34	4.55
trait anxiety [STAI]	46.82	5.76
depressive symptoms [PHQ-2]	1.54	1.38

### Questionnaires

#### Anxiety Sensitivity Index-3 (ASI-3)

The ASI-3^28^ is an 18-item (e.g., “When I feel pain in my chest, I worry that I’m going to have a heart attack”) measure that assesses the tendency to fear anxiety-related sensations^29^ on a 5-point Likert scale ranging from 0 (very little) to 4 (very much). In previous studies, the ASI-3 has demonstrated good reliability and validity (e.g., internal consistency α = 0.92^28,30,31^). The current study also demonstrated excellent internal consistency for the ASI-3 (i.e., α = 0.93).

#### State-Trait Anxiety Inventory (STAI)

The trait portion of the STAI^32,33^ measures the general proneness to experience anxiety and perceive situations as threatening with 20 items (e.g., “I feel nervous and restless”) on a 4-point Likert scale. The STAI has demonstrated good reliability and validity (e.g., internal consistency α = 0.86^32^). The current study also demonstrated good internal consistency for the trait version of the STAI (i.e., α = 0.94).

#### Generalized Anxiety Disorder-2 (GAD-2)

The GAD-2^34,35^ is a 2-item (e.g., “Feeling nervous, anxious or on edge”) measure that assesses generalized anxiety symptoms during the last two weeks on a 4-point Likert scale ranging from 0 (not at all) to 3 (nearly every day). The GAD-2 demonstrated a high sensitivity for diagnosing anxiety disorders. The current study demonstrated an acceptable internal consistency for the GAD-2 (i.e., α = 0.82).

#### Patient Health Questionnaire-2 (PHQ-2)

The PHQ-2^36,37^ is a 2-item (e.g., “Little interest or pleasure in doing things”) measure that assesses depressive symptomatology during the last two weeks on a 4-point Likert scale ranging from 0 (not at all) to 3 (nearly every day). The PHQ-2 demonstrated a high sensitivity for diagnosing depressive disorders. The current study demonstrated good internal consistency for the PHQ-2 (i.e., α = 0.79).

#### Illness Attitude Scale (IAS)

The IAS is a 29-item (e.g., “Do you worry about your health”) measure assessing hypochondriacal fears and beliefs on a 5-point scale ranging from 0 (no) to 4 (most of the time). The IAS comprised 9 subscales: (I) worry about illness, (II) concerns about pain, (III), health habits, (IV) hypochondriacal beliefs, (V) thanatophobia (fear of death), (VI) disease phobia, (VII) bodily preoccupations, (VIII) treatment experience, and (IX) effects of symptoms. In previous studies, the IAS demonstrated good to excellent reliability and validity (e.g., test–retest reliability r = 0.89^38^). The current study demonstrated an excellent internal consistency for the IAS (i.e., α = 0.89).

#### Patient Health Questionnaire 15 (PHQ-15)

The PHQ-15 is a 15-item measure assessing somatization and somatic symptom severity for the last four weeks using the most prevalent somatic symptoms of the DSM-IV somatization disorder (e.g., back pain, headache). The items were rated on a 3-point Likert scale ranging from 0 (“not bothered at all”) to 2 (“bothered a lot”). The PHQ-15 demonstrated good reliability and validity (e.g., α = 0.80^39^). The current study demonstrated an acceptable internal consistency for the PHQ-15 (i.e., α = 0.77).

### Experimental stimuli

The online experiment including ratings, timing, and presentation of stimuli was realized using an online survey platform (soscisurvey.de).

#### Imagery scripts

Fifteen narrative imagery scripts were used. Scripts were categorized into the following categories: neutral (e.g., loading the dishwasher), standard fear (e.g., attack by a snake or a stranger), being told potentially bad news about having a serious illness, signs of a serious illness, and experiencing illness-related symptoms. Following previous studies, we used three scripts per category^9,17,40^. Scripts comprised between 28 and 44 words. All scripts were developed according to the recommendations of Lang^41^. Thus, all scripts were written in first person present tense and included sensory and context information as well as behavioral and somato-visceral responses. The English translation of all scripts is provided in Table 2.

**Table 2:** Neutral, standard fear, body symptom and illness-related scripts.

Neutral scene
I run the comb through my damp hair, check the fit of my clothes. "Everything fits." The water runs down the drain. I turn off the tap and go
I take the groceries out of the car. I pick up the shopping bag, press it against my chest tightly, and lean over to close the trunk. What am I going to eat today?
I put a plate into the full dishwasher. “Now I can turn it on”. I put the dishwasher tablet in the dishwasher. There’s a soft beep as I switch it on
**Standard fear scene**
I suddenly wake up in my sleeping bag. It's pitch-black outside and I feel a snake glide up my legs. I scream, trying to get out of the sleeping bag. Will it bite me?
I am alone in a deserted area when suddenly a man with a knife approaches me and smirks menacingly. I run faster. My heart is pounding in my chest. What is he going to do to me?
I hear the screeching of brakes. I look up and see my girlfriend has been hit by a car. Her leg is crushed, a vein is torn, blood pumps onto the street. I can't think clearly, how am I supposed to help her?
**Getting bad news about having a serious illness**
I have another doctor's appointment because of my complaints, I'm sitting in the waiting room. What if the doctor has found something? My heart is beating faster, I am starting to sweat. Maybe it's cancer? How am I going to live with that? I'm sitting in my kitchen at home, holding my phone. It rings. It is the doctor who wants to tell me the results of the last examination. I am tense, my hands start to shake. What if I have something wrong with my heart? I am called into the doctor's office to go into the treatment room. I get nervous, my hands sweat, I feel sick. He might tell me that I have cancer and that I am going to die. How should I go on then?
**Signs of serious disease**
My stomach cramps uncomfortably. I feel my stomach carefully. There is something wrong with my body, what is happening to me? Do I have stomach cancer? This is going to be the end of me I am working on my computer. Suddenly I feel an unpleasant tingling sensation in my hands and feet. I feel very unwell. Is this typical of MS? How am I supposed to go on with my life if I have this? I'm sitting on the couch and I feel that tug behind my forehead again. I touch my temple, my whole body is tense, I am scared. Could the doctor have missed something? Could it be a tumor? Am I going to die?
**Experiencing body symptoms**
I lie comfortably on the couch. There they are again, the headaches. I touch my forehead. I feel hot and cold, my heart beats faster. I hope it will be over soon I'm at work. My stomach starts to cramp and I feel sick. It feels different than usual. I get nervous, my hands get sweaty. What is it now? I try to take it easy I am out for a walk with my family. I suddenly feel dizzy. I try to stay calm, but I can't control my nervousness. I am afraid. Why is this happening again? I hope the doctor can explain!

#### Visual stimuli

A blue circle signaled participants to vividly imagine the scenes.

#### Ratings

Participants were asked to rate the vividness of imagery (1 = not vivid at all, 9 = very vivid) as well as their experienced anxiety (1 = no anxiety, 9 = very strong anxiety), displeasure (1 = pleasant, 9 = unpleasant), emotional arousal (1 = relaxed, 9 = aroused) and the wish to avoid imagery (1 = no wish to avoid, 9 = strong wish to avoid imagery) during the imagery phase on a 9-point rating scale.

### Procedure

The study procedure followed well-established protocols for mental imagery^9,17,23,40^: Participants were instructed to read the scripts and, then, during the presentation of the blue circle, to vividly imagine the scenes, as if they were actively involved in the scene. Script texts were presented on screen for fixed time period of 12 s, immediately followed by a 12 s imagery phase. After each imagery, participants were asked to rate the vividness of imagery as well as their experienced unpleasantness, emotional arousal, anxiety, and avoidance tendency during the imagery phase. Scripts were presented in a pseudo-randomized order with the restriction that no more than two scripts of the same content category were presented consecutively. To familiarize participants with the imagery and rating procedure, one trial was presented before the start of the experiment, which included the reading of a neutral script as well as an imagery and rating phase. After completing the experimental task, participants were asked to indicate whether they experienced any interruptions ('Were you interrupted during the study?'), disturbances ('Were you disturbed during the study?'), or distractions ('Were you distracted during the study?') during the experiment using a dichotomous scale (yes/no). Finally, participants were asked to complete the questionnaires mentioned above.

### Data analyses

In line with previous studies^9,16,17,23^, all ratings were averaged per category. All statistical models were built to align with our theoretical considerations and formulated hypotheses. First, the effects of HA on differences in emotional responses during imagery between script categories were analyzed using mixed effects regression models with the repeated measurement factor *category* (neutral vs. standard fear vs. getting bad news vs. signs of serious illness vs. body symptoms; Level 1) nested within persons (Level 2). The category factor was dummy coded with neutral scripts as the reference category. Moreover, the continuous between-subjects predictor HA as well as the interaction between HA and category was included in the model. To probe significant interactions, exploratory simple slope analyses were run^42^ to evaluate the significance of the category effects on emotional responses for conditional values of HA, i.e., for low (5% percentile, IAS = 6), moderate (50% percentile, IAS = 20), and severe (95% percentile, IAS = 49) HA. We set the threshold for severe HA at the 95th percentile (IAS score of 49), in line with Hedman et al.^43^, that determined an IAS score of 47 as the cutoff for severe HA. We calculated difference scores by subtracting emotional responses to each imagery category from those in the neutral condition. In all simple slope analyses, category was dummy coded with standard fear scripts as the reference category. Second, to test for the specificity of the interaction between HA and emotional responses to illness-related imagery, we included other relevant predictors (i.e., anxiety sensitivity, trait anxiety, somatic symptom severity, depressive and anxiety symptoms) and the interactions between the respective predictors and the factor category in the respective models. Models were fitted for each outcome separately. All analyses were adjusted for age and gender as well as multiple testing (alpha level was set at 0.01). All models included a random intercept and applied a restricted maximum likelihood estimation (REML). All predictors and outcomes were standardized. Correlations among predictors were < 0.7 and indices of multicollinearity were acceptable (all VIF < 10^44^), suggesting a lack of redundancy in model predictors^45^. Checks of the assumptions underlying the linear mixed effects models indicated no substantial violations, negating the need for any specific measures beyond the initial procedures implemented. We used BIC and AIC indices to evaluate the fit of the models. Our analyses revealed that the parsimonious model, testing the effect of health anxiety on differences in emotional responses during imagery between script categories, emerged as the best-fitting model according to BIC and AIC. Specifically, the introduction of additional predictors did not lead to a proportional improvement in model fit. In fact, these models demonstrated higher AIC and BIC values (∆BIC and ∆AIC > 10^46^), suggesting that the increased complexity did not enhance the model's explanatory power. This indicates that the inclusion of other factors, beyond health anxiety, did not significantly contribute to explaining the variance in fearful imagery, which is in line with our hypothesis. Statistical analyses were conducted with jamovi (version 2.3.).

## Results

### Affective modulation by imagery of illness-related and fear scenes

Table 3 summarizes the means and standard deviations of reported anxiety, aversion, emotional arousal, avoidance tendency, and vividness to imagery of illness-related, fear, and neutral narrative scenes. Participants reported more anxiety, aversion, emotional arousal, and a stronger tendency to avoid imagery during mental imagery of fear and illness-related scenes (experiencing body symptoms, signs of a serious illness, and potentially getting bad news about having a life-threatening disease) than during imagery of neutral scenes, all *p*-values < 0.001 (see Supplementary Tables S1–S4). Illness-related imagery was rated as less vivid than the neutral scenes, all *p*-values < 0.001, while no evidence was found for differences in imagery vividness between fear and neutral scenes, *p* = 0.216 (see Supplementary Table S5).

**Table 3:** Means and standard deviations of reported anxiety, valence, arousal, avoidance tendency and vividness to imagery of illness-related, fear and neutral narrative scripts.

Script category	Anxiety		Valence		Arousal		Avoidance		Vividness	
	*M*	*SD*	*M*	*SD*	*M*	*SD*	*M*	*SD*	*M*	*SD*
Neutral	1.40	0.88	2.70	1.17	2.47	1.13	1.46	0.91	7.29	1.54
Standard fear	6.90	1.87	8.04	1.03	7.85	1.13	7.07	2.13	7.14	1.37
Signs of severe disease	4.90	2.01	6.77	1.23	6.09	1.50	5.41	2.28	5.85	1.84
Receiving bad news	5.47	1.99	7.00	1.16	6.65	1.47	5.81	2.40	6.35	1.81
Body symptoms	4.41	1.78	6.91	1.08	5.95	1.27	5.47	2.11	6.58	1.68

Verbal anchors: Valence: 1 = pleasant, 9 = unpleasant; Arousal: 1 = relaxed, 9 = aroused; Anxiety: 1 = no anxiety, 9 = severe anxiety, Avoidance: 1 = no wish to avoid, 9 = strong wish to avoid imagery, Vividness: 1 = not at all vivid, 9 = very vivid imagery.

### Effects of HA on emotional responses to mental imagery

**Figure 1:**
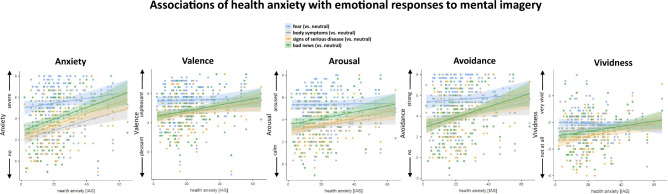
Association of health anxiety (indexed by the illness anxiety inventory) with reported anxiety, displeasure, emotional arousal, wish to avoid imagery and vividness during imagery of standard threat, and illness-related narrative scenes (i.e., experiencing body symptoms, signs of a severe disease, and receiving bad news about having a severe disease). Responses were standardized relative to the respective responses to imagery of neutral scenes. Regression lines represent linear fits to the observed data (dots). Shaded areas represent the standard errors of the regressions.

The observed emotional modulation significantly varied by the level of HA, for anxiety: HA × Category *F*(4, 560) = 9.39, *p* < 0.001; displeasure: HA × Category *F*(4, 560) = 4.75, *p* < 0.001, arousal: HA × Category *F*(4, 560) = 5.66, *p* < 0.001; avoidance tendency: HA × Category *F*(4, 560) = 6.88, *p* < 0.001; vividness: HA × Category , *F*(4, 560) = 7.11, *p* < 0.001. As depicted in Fig. 1, higher HA was associated with reports of higher anxiety, aversion, emotional arousal, a stronger avoidance tendency, and vividness during vivid imagination of illness-related (signs of serious illness or potentially getting bad news about having a life-threatening disease) relative to neutral narrative scenes, all *p*-values < 0.05 (for parameter estimates from mixed effects regression models see Supplementary Tables S1–S5). For imagery of scenes including body symptoms without providing further illness-related concerns or context information, HA was only related to higher anxiety and avoidance tendencies (*p*-values < 0.05), but not to emotional arousal, displeasure, and vividness during imagery (*p*-values > 0.05). In contrast, HA did not modulate emotional responses to imagery of fear vs. neutral scenes, all *p*-values > 0.05 (see Supplementary Tables S1–S5).

Exploratory simple slope analyses were run to compare fear responses to imagery scripts for conditional values of the between-subjects predictor HA. Participants with low and moderate HA reported much less anxiety, displeasure, emotional arousal, vividness, and a less pronounced avoidance tendency during imagery of illness-related than standard fear scenes, all *p*-values < 0.05 (see Supplementary Tables S6–S10). In contrast, participants with severe HA (IAS = 49; 95% percentile), showed comparably high levels of anxiety, aversion, arousal, vividness, and avoidance tendencies to mental imagery of potentially getting bad news about having a life-threatening disease and survival threat scenes (i.e., standard fear scenes) as compared to neutral scenes, all *p*-values > 0.05. As observed for individuals with low and moderate HA, imagery of signs of a serious illness or experiencing illness-related symptoms elicited slightly lower emotional responses than the standard fear scripts for those with severe HA (*p*-values < 0.001; except for avoidance tendency during imagery of signs of severe disease, *p* = 0.08; see Supplementary Tables S6–S10).

### Multiple predictors for affective modulation by illness-related imagery

Trait anxiety, anxiety sensitivity, somatic symptom severity, and depressive and anxiety symptoms had no significant effect on the affective modulation to illness-related mental imagery, all p-values > 0.05 (see Table 4 for results of the fixed effect omnibus tests of the interactions). Importantly, in models incorporating these additional predictors, reported anxiety, displeasure, emotional arousal, and avoidance tendencies to imagery of illness-related scenes were still varied by HA, all *p*-values < 0.05 (see Table 4; for parameter estimates from mixed effects regression models see also Supplementary Tables S11–S15). However, vividness of imagery was no longer modulated by HA, *p* = 0.089.

**Table 4:** Fixed effect omnibus tests of the interactions between imagery category and the respective predictors (i.e., health anxiety, anxiety sensitivity, somatic symptom severity, depressive and anxiety symptoms, and trait anxiety).

By category interactions	Anxiety		Arousal		Valence		Avoidance		Vividness	
	*F*_1,540_	*P*	*F*_1,540_	*p*	*F*_1,540_	*p*	*F*_1,540_	*p*	*F*_1,540_	*p*
Health anxiety	5.52	0.001	5.62	0.001	5.50	0.001	4.90	0.003	3.01	0.089
Anxiety sensitivity	0.29	1.0	1.61	0.850	0.70	1.0	0.38	1.0	0.77	1.0
Somatic symptom severity	0.38	1.0	2.30	0.289	0.76	1.0	0.50	1.0	1.09	1.0
Depressive symptoms	2.10	0.395	0.55	1.0	1.20	1.0	1.72	0.721	0.31	1.0
Anxiety symptoms	0.34	1.0	1.12	1.0	0.74	1.0	0.03	1.0	0.28	1.0
Trait anxiety	0.16	1.0	0.37	1.0	0.32	1.0	0.40	1.0	0.41	1.0

### Sensitivity analysis

To examine differences between included and excluded participants, all models were re-run by including group (included vs. excluded participant) as a between-subjects factor. The analyses revealed that the observed response pattern did not differ between participants who were included or excluded from the analyses, all by-group interactions *p* > 0.05. Moreover, participants did not differ regarding sociodemographic and questionnaire data, *p* > 0.05. To ensure the robustness of our results, we conducted a sensitivity analysis by including the full sample (*N* = 175), which encompasses participants who reported interruptions, disturbances, or distractions during the experiment. This analysis confirmed that the inclusion of these participants did not alter the outcomes as reported above, thereby corroborating the consistency and reliability of our reported findings.

## Discussion

In the present study, we aimed to examine (a) the relationship between HA and fear responses to mental images centered around having or getting a potential illness, (b) whether the mere imagination of actually innocuous body symptoms that could be related to illnesses elicit stronger fear responses in individuals with higher HA, and (c) whether these associations are specific for HA and illness-related images. To this end, we used a well-controlled script-driven imagery task, systematically comparing emotional responses to disease-related imagery with neutral and standard fear imagery. All participants showed a stronger fear response to imagery of standard fear and illness-related scenes compared to neutral mental imagery. The fearful response to illness-related imagery was, however, more pronounced in individuals with higher HA. No such modulation by HA was found for imagery of fear scripts. Importantly, associations with imagery-induced affective modulation were specific for HA and could not be better explained by other psychological factors such as anxiety sensitivity or somatic symptom severity.

Lang's^41,47^ bio-informational theory of emotional imagery posits that when individuals engage in mental imagery of emotional stimuli, such as threat or early indicators of threat (e.g. interoceptive cues), it activates an associative network that overlaps with the network activated during the actual experience of the stimuli^41,48^. Accordingly, it has been demonstrated that mental imagery of aversive scenes triggers defensive reflex and autonomous-sympathetic mobilization along with verbal reports of fear and displeasure^9–11,23,24^ paralleling response pattern observed during anticipation or real-life encounters of a threat^49–53^, with a similar threat circuitry mediating theses fear responses^54–57^. The present study indicates that mental imagery of narrative scenes centered around concerns commonly reported in individuals with HA also activates such an associative fear network as evidenced by elevated fear responses to these mental images. This increased fear response is suggested to result from increased associative strength within an elaborated fear network related to the imagined aversive stimulus^41,58^.

The current findings suggest that individuals with higher HA may have a more elaborated illness-specific fear memory network, which might explain their increased fearful responding during illness-related imagery^48,58^. This corresponds with a recent study that demonstrated that HA is associated with a more pronounced fear response to imagery of contracting COVID-19 during the COVID-19 pandemic^16^. Interestingly, in those individuals with severe (vs. mild) HA, mental images of potentially getting bad news about having a life-threatening disease elicited a comparable fear response as survival threat scenes, suggesting a sensitization of brain's fear system to these images in pathological HA^11^. A similar pattern has also been demonstrated in patients with anxiety disorders who exhibited a comparable defensive activation during mental imagery of disorder-specific (e.g. social or panic-threatening scenes) and survival threat scenes which was not observed in healthy controls^11,12,22,59^. Thus, these illness-related mental images may in particular result into maladaptive worry, anxiety and behaviors (e.g., checking behavior, hypervigilance to body symptoms) in individuals with pathological HA. Moreover, this finding aligns with evidence on interpretation bias in health anxiety in that individuals may misinterpret body symptoms as signs of a threatening illness, demonstrating a bias comparable in magnitude to non-health-related interpretation biases^19^. Such biases may contribute to the heightened fear response to health-related threat imagery observed in our study and thus may further perpetuate the cycle of maladaptive worry, anxiety, and behaviors in those with pathological HA.

Moreover, our study provides preliminary evidence that mental imagery of the occurrence of bodily symptoms indicative of a potential illness elicits stronger fear reactions, particularly in individuals exhibiting elevated HA levels. This is consistent with a recent study demonstrating that adolescents with chronic pain exhibited a more pronounced fear response to imagery of pain-associated body symptoms^60^. Of importance, the narrative scenes that focused on the experience of body symptoms did not include any interpretations or concerns relating these symptoms to a serious disease. Based on the bio-informational theory^41^, one can suggest that body symptoms might activate an associative network including associations to semantic information (i.e., meaning information) about the threatening nature of these symptoms (e.g., danger, serious illness) as well as to response information (i.e., somato-visceral and behavioral responses). Thus, exaggerated fearful imagery in individuals with increased levels of HA may result from greater associative fear network activation based on high interconnection and associative strength between body symptoms and threat-related perceptual, and semantic information as well as somatovisceral and preparatory motor responses^23,41,48^. Indeed, future studies ought to investigate whether imagery of body symptoms actually prompts the hypothesized increased activation of the brain’s fear network in individuals with high HA, albeit studies support this assumption by demonstrating that individuals with fear of body symptoms exhibit a stronger activation of the brain's defensive circuit during anticipation or imagery of feared bodily symptoms^49,51,56,61^.

The present findings indicate that elevated fear responses to illness-related imagery are uniquely linked to HA and cannot be better explained by other psychological factors such as trait anxiety, anxiety sensitivity, somatic symptom severity, or depressive and anxiety symptoms. Thus, fearful imagery of illness-related scenes might be a specific correlate of increased HA which is supported by the relatively high prevalence of recurrent and distressing mental images of getting or having life-threatening diseases in individuals with pathological HA^8,62^. Our study revealed that the vividness of these images was particularly increased among those with elevated HA which corroborate evidence that narrative scenes closely related to an individual's specific fears or disorders are imagined as more vivid^9^. This heightened vividness of illness-related threat scenes may further intensify anxiety and avoidance tendencies during imagery. In the present study, increased vividness and fear during imagery was accompanied by a stronger tendency to avoid mental images related to diseases and body symptoms, indicating that persons with high HA are prone to engage in maladaptive behaviors to avoid such imagery. These maladaptive behaviors, including avoidance, suppression, distraction, reassurance or safety behavior to terminate or attenuate these aversive images, may in turn perpetuate pathological health worries, mental images, and fear, thus contributing to the persistence and chronicity of pathological HA^1,8,13,15^. Thus, fearful imagery of potentially receiving a diagnosis of a life-threatening illness or encountering physical symptoms potentially plays a significant role in the emergence and perpetuation of HA.

While our study provides important insights into the relationship between fearful imagery and HA, some limitations should be considered. In this study, our assessment of defensive motivational activation was limited to verbal indicators. To obtain a more comprehensive characterization of fear processing during mental imagery of illness-related narrative scenes, future studies should incorporate measurements of behavioral and physiological correlates of fear, such as the mobilization of defensive reflexes and autonomic arousal. Furthermore, it is important to note that our sample consisted predominantly of young, female participants, reported to have a prevalence of 4.15% for severe health anxiety^63^. This contrasts with the higher prevalence of 5.02% in middle-aged females and lower rates in men and younger females^63^. Although our analyses were corrected for age and gender, suggesting consistency in the findings across various groups, the specific demographic prevalence rates indicate potential limits to generalizability of our findings across different demographic groups. To provide a more robust evaluation of the relationship between fearful imagery and HA, future studies ought to replicate these findings using larger and more diverse samples in terms of age, gender, and psychopathology. Due to the cross-sectional design of our study, we are unable to ascertain the directionality of the relationship between fearful imagery and HA, i.e., whether fearful imagery influences HA or vice versa. Thus, longitudinal studies are imperative to unfold the temporal associations between fearful imagery, maladaptive behaviors, and the trajectory of HA over time.

## Conclusion

The results of the study document that individuals with high levels of HA show a pronounced fear response to mental images related to themes commonly reported by individuals with severe HA, such as signs of a serious illness or potentially getting bad news about having a life-threatening disease. Therefore, the present findings indicate that imagery of specific cues and contexts related to potential illness can activate an elaborated illness-related fear memory network, particularly in those individuals with high levels of HA. Specifically targeting these intrusive images and the related illness-specific fear memory network via imagery exposure, imagery re-scripting, or interoceptive exposure could enhance and refine the treatment of severe HA^64–68^. Exposure-based treatments and imagery re-scripting have been proven to be effective in reducing disorder severity as well as fearful imagery in anxiety disorders (e.g., social and generalized anxiety disorders, post-traumatic stress disorder, specific phobias)^69–72^. Preliminary evidence from a non-randomized pilot study indicates that imagery re-scripting in individuals with severe HA significantly reduced health anxiety as well as vividness, distress and frequency of intrusive images^73^. In future studies, the assessment of fear responses toward illness-related imagery might help to effectively target intrusive images and the related illness-specific fear memory network but also, to monitor treatment outcomes. Since we found that imagery related to illness-specific scenarios is uniquely associated with health anxiety and not accounted for by other (clinical) psychological constructs, such imagery could serve as a distinctive marker and potential diagnostic tool for HA. Moreover, script-driven imagery may provide a productive methodology in future studies for characterizing fear response patterns and its neurobiological correlates of HA. Future longitudinal studies are essential to provide more insights into the temporal dynamics of fearful imagery and HA, and to help elucidate the role of fearful imagery in the development or proliferation of HA. Given that health anxiety is prevalent in various health conditions and diagnoses, such as illness anxiety disorder and panic disorder, the present findings could have broader implications for understanding and treating anxiety-related symptoms across a spectrum of clinical presentations, warranting future research to investigate the impact of illness-related images across these populations.

### Supplementary Information

Supplementary Tables.

## Data Availability

The data, that support the findings of this study, are available from the corresponding author (christoph.benke@uni-marburg.de) upon reasonable request.
