# Supplementary material for: Health anxiety amplifies fearful responses to illness-related imagery
Source: Sci Rep. 2024 Feb 22;14:4345. doi: 10.1038/s41598-024-54985-y (PMC10883981; doi:10.1038/s41598-024-54985-y)
Supplement: Supplementary file 1 — Supplementary Tables. [file 41598_2024_54985_MOESM1_ESM.docx]

**Supplementary Materials**

**Health anxiety amplifies fearful responses to illness-related imagery**

Christoph Benke, Ph.D., Laura-Marie Wallenfels, B.Sc., Gaby M. Bleichhardt, Ph.D.,

& Christiane A. Melzig, Ph.D.

**Supplementary results**

***Table S1.*** Fixed Effects Parameter Estimates of mixed effects regression models examining the effect of mental imagery content and HA on reported anxiety.

|  | | | | | | | | | | | | | | | | | |
| --- | --- | --- | --- | --- | --- | --- | --- | --- | --- | --- | --- | --- | --- | --- | --- | --- | --- |
|  | | | | | | | | **99% Confidence Interval** | | | |  | | | | | |
|  | | **Effect** | | **Estimate** | | **SE** | | **Lower** | | **Upper** | | **df** | | **t** | | **p** | |
|  |  | (Intercept) |  | -1.28 |  | 0.05 |  | -1.42 |  | -1.14 |  | 364.97 |  | -23.29 |  | < .001 |  |
|  |  | body symptoms - neutral |  | 1.19 |  | 0.06 |  | 1.05 |  | 1.34 |  | 560.00 |  | 21.21 |  | < .001 |  |
|  |  | signs of severe disease - neutral |  | 1.39 |  | 0.06 |  | 1.24 |  | 1.53 |  | 560.00 |  | 24.70 |  | < .001 |  |
|  |  | standard fear - neutral |  | 2.18 |  | 0.06 |  | 2.04 |  | 2.33 |  | 560.00 |  | 38.77 |  | < .001 |  |
|  |  | bad news - neutral |  | 1.62 |  | 0.06 |  | 1.47 |  | 1.76 |  | 560.00 |  | 28.72 |  | < .001 |  |
|  |  | health anxiety [IAS] |  | 0.01 |  | 0.06 |  | -0.13 |  | 0.16 |  | 355.86 |  | 0.25 |  | 1.000 |  |
|  |  | age |  | -0.06 |  | 0.04 |  | -0.17 |  | 0.05 |  | 138.00 |  | -1.49 |  | 0.690 |  |
|  |  | gender |  | 0.08 |  | 0.04 |  | -0.03 |  | 0.19 |  | 138.00 |  | 1.95 |  | 0.265 |  |
|  |  | body symptoms - neutral ✻ health anxiety [IAS] |  | 0.23 |  | 0.06 |  | 0.09 |  | 0.38 |  | 560.00 |  | 4.11 |  | < .001 |  |
|  |  | signs of severe disease - neutral ✻ health anxiety [IAS] |  | 0.28 |  | 0.06 |  | 0.13 |  | 0.42 |  | 560.00 |  | 4.96 |  | < .001 |  |
|  |  | standard fear - neutral ✻ health anxiety [IAS] |  | 0.12 |  | 0.06 |  | -0.03 |  | 0.26 |  | 560.00 |  | 2.08 |  | 0.190 |  |
|  |  | bad news - neutral ✻ health anxiety [IAS] |  | 0.28 |  | 0.06 |  | 0.14 |  | 0.43 |  | 560.00 |  | 5.03 |  | < .001 |  |
|  | | | | | | | | | | | | | | | | | |

***Table S2.*** Fixed Effects Parameter Estimates of mixed effects regression models examining the effect of mental imagery content and HA on reported displeasure.

|  | | | | | | | | | | | | | | | | | |
| --- | --- | --- | --- | --- | --- | --- | --- | --- | --- | --- | --- | --- | --- | --- | --- | --- | --- |
|  | | | | | | | | **99% Confidence Interval** | | | |  | | | | | |
|  | | **Effect** | | **Estimate** | | **SE** | | **Lower** | | **Upper** | | **df** | | **t** | | **p** | |
|  |  | (Intercept) |  | -0.00 |  | 0.03 |  | -0.07 |  | 0.07 |  | 138.00 |  | -0.00 |  | 1.000 |  |
|  |  | body symptoms - neutral |  | 1.94 |  | 0.05 |  | 1.81 |  | 2.07 |  | 560.00 |  | 37.75 |  | < .001 |  |
|  |  | signs of severe disease - neutral |  | 1.88 |  | 0.05 |  | 1.74 |  | 2.01 |  | 560.00 |  | 36.48 |  | < .001 |  |
|  |  | standard fear - neutral |  | 2.46 |  | 0.05 |  | 2.33 |  | 2.60 |  | 560.00 |  | 47.92 |  | < .001 |  |
|  |  | bad news - neutral |  | 1.98 |  | 0.05 |  | 1.85 |  | 2.12 |  | 560.00 |  | 38.55 |  | < .001 |  |
|  |  | health anxiety [IAS] |  | 0.08 |  | 0.03 |  | 0.01 |  | 0.16 |  | 138.00 |  | 3.02 |  | 0.015 |  |
|  |  | gender |  | 0.09 |  | 0.06 |  | -0.07 |  | 0.25 |  | 138.00 |  | 1.48 |  | 0.700 |  |
|  |  | age |  | -0.01 |  | 0.00 |  | -0.01 |  | -0.00 |  | 138.00 |  | -2.92 |  | 0.020 |  |
|  |  | body symptoms - neutral ✻ health anxiety [IAS] |  | 0.12 |  | 0.05 |  | -0.01 |  | 0.25 |  | 560.00 |  | 2.36 |  | 0.095 |  |
|  |  | signs of severe disease - neutral ✻ health anxiety [IAS] |  | 0.16 |  | 0.05 |  | 0.03 |  | 0.29 |  | 560.00 |  | 3.13 |  | 0.010 |  |
|  |  | standard fear - neutral ✻ health anxiety [IAS] |  | 0.03 |  | 0.05 |  | -0.10 |  | 0.17 |  | 560.00 |  | 0.66 |  | 1.000 |  |
|  |  | bad news - neutral ✻ health anxiety [IAS] |  | 0.18 |  | 0.05 |  | 0.05 |  | 0.31 |  | 560.00 |  | 3.52 |  | < .001 |  |
|  | | | | | | | | | | | | | | | | | |

***Table S3.*** Fixed Effects Parameter Estimates of mixed effects regression models examining the effect of mental imagery content and HA on reported arousal.

|  | | | | | | | | | | | | | | | | | |
| --- | --- | --- | --- | --- | --- | --- | --- | --- | --- | --- | --- | --- | --- | --- | --- | --- | --- |
|  | | | | | | | | **99% Confidence Interval** | | | |  | | | | | |
|  | | **Effect** | | **Estimate** | | **SE** | | **Lower** | | **Upper** | | **df** | | **t** | | **p** | |
|  |  | (Intercept) |  | -0.00 |  | 0.04 |  | -0.09 |  | 0.09 |  | 138.00 |  | -0.00 |  | 1.000 |  |
|  |  | body symptoms - neutral |  | 1.57 |  | 0.05 |  | 1.44 |  | 1.70 |  | 560.00 |  | 31.75 |  | < .001 |  |
|  |  | signs of severe disease - neutral |  | 1.63 |  | 0.05 |  | 1.50 |  | 1.76 |  | 560.00 |  | 32.99 |  | < .001 |  |
|  |  | standard fear - neutral |  | 2.42 |  | 0.05 |  | 2.30 |  | 2.55 |  | 560.00 |  | 49.06 |  | < .001 |  |
|  |  | bad news - neutral |  | 1.88 |  | 0.05 |  | 1.76 |  | 2.01 |  | 560.00 |  | 38.11 |  | < .001 |  |
|  |  | health anxiety [IAS] |  | 0.11 |  | 0.04 |  | 0.02 |  | 0.21 |  | 138.00 |  | 3.18 |  | 0.010 |  |
|  |  | gender |  | 0.14 |  | 0.08 |  | -0.07 |  | 0.34 |  | 138.00 |  | 1.73 |  | 0.430 |  |
|  |  | age |  | -0.01 |  | 0.00 |  | -0.02 |  | -0.00 |  | 138.00 |  | -3.06 |  | 0.015 |  |
|  |  | body symptoms - neutral ✻ health anxiety [IAS] |  | 0.13 |  | 0.05 |  | -0.00 |  | 0.25 |  | 560.00 |  | 2.56 |  | 0.055 |  |
|  |  | signs of severe disease - neutral ✻ health anxiety [IAS] |  | 0.17 |  | 0.05 |  | 0.04 |  | 0.30 |  | 560.00 |  | 3.46 |  | < .001 |  |
|  |  | standard fear - neutral ✻ health anxiety [IAS] |  | 0.01 |  | 0.05 |  | -0.12 |  | 0.14 |  | 560.00 |  | 0.24 |  | 1.000 |  |
|  |  | bad news - neutral ✻ health anxiety [IAS] |  | 0.17 |  | 0.05 |  | 0.04 |  | 0.29 |  | 560.00 |  | 3.34 |  | < .001 |  |
|  | | | | | | | | | | | | | | | | | |

***Table S4.*** Fixed Effects Parameter Estimates of mixed effects regression models examining the effect of mental imagery content and HA on reported avoidance tendency.

|  | | | | | | | | | | | | | | | | | |
| --- | --- | --- | --- | --- | --- | --- | --- | --- | --- | --- | --- | --- | --- | --- | --- | --- | --- |
|  | | | | | | | | **99% Confidence Interval** | | | |  | | | | | |
|  | | **Effect** | | **Estimate** | | **SE** | | **Lower** | | **Upper** | | **df** | | **t** | | **p** | |
|  |  | (Intercept) |  | -0.00 |  | 0.05 |  | -0.13 |  | 0.13 |  | 138.00 |  | -0.00 |  | 1.000 |  |
|  |  | body symptoms - neutral |  | 1.44 |  | 0.05 |  | 1.30 |  | 1.58 |  | 560.00 |  | 26.51 |  | < .001 |  |
|  |  | signs of severe disease - neutral |  | 1.42 |  | 0.05 |  | 1.28 |  | 1.56 |  | 560.00 |  | 26.12 |  | < .001 |  |
|  |  | standard fear - neutral |  | 2.02 |  | 0.05 |  | 1.88 |  | 2.16 |  | 560.00 |  | 37.12 |  | < .001 |  |
|  |  | bad news - neutral |  | 1.56 |  | 0.05 |  | 1.42 |  | 1.71 |  | 560.00 |  | 28.75 |  | < .001 |  |
|  |  | health anxiety [IAS] |  | 0.17 |  | 0.05 |  | 0.04 |  | 0.30 |  | 138.00 |  | 3.37 |  | < .001 |  |
|  |  | gender |  | 0.12 |  | 0.11 |  | -0.17 |  | 0.40 |  | 138.00 |  | 1.06 |  | 1.000 |  |
|  |  | age |  | -0.00 |  | 0.00 |  | -0.01 |  | 0.01 |  | 138.00 |  | -0.57 |  | 1.000 |  |
|  |  | body symptoms - neutral ✻ health anxiety [IAS] |  | 0.15 |  | 0.05 |  | 0.01 |  | 0.29 |  | 560.00 |  | 2.74 |  | 0.030 |  |
|  |  | signs of severe disease - neutral ✻ health anxiety [IAS] |  | 0.21 |  | 0.05 |  | 0.07 |  | 0.35 |  | 560.00 |  | 3.93 |  | < .001 |  |
|  |  | standard fear - neutral ✻ health anxiety [IAS] |  | 0.06 |  | 0.05 |  | -0.08 |  | 0.20 |  | 560.00 |  | 1.02 |  | 1.000 |  |
|  |  | bad news - neutral ✻ health anxiety [IAS] |  | 0.23 |  | 0.05 |  | 0.09 |  | 0.37 |  | 560.00 |  | 4.31 |  | < .001 |  |
|  | | | | | | | | | | | | | | | | | |

***Table S5.*** Fixed Effects Parameter Estimates of mixed effects regression models examining the effect of mental imagery content and HA on reported vividness.

|  | | | | | | | | | | | | | | | | | |
| --- | --- | --- | --- | --- | --- | --- | --- | --- | --- | --- | --- | --- | --- | --- | --- | --- | --- |
|  | | | | | | | | **99% Confidence Interval** | | | |  | | | | | |
|  | | **Effect** | | **Estimate** | | **SE** | | **Lower** | | **Upper** | | **df** | | **t** | | **p** | |
|  |  | (Intercept) |  | 0.00 |  | 0.06 |  | -0.16 |  | 0.16 |  | 138.00 |  | 0.00 |  | 1.000 |  |
|  |  | body symptoms - neutral |  | -0.41 |  | 0.07 |  | -0.60 |  | -0.23 |  | 560.00 |  | -5.65 |  | < .001 |  |
|  |  | signs of severe disease - neutral |  | -0.84 |  | 0.07 |  | -1.02 |  | -0.65 |  | 560.00 |  | -11.40 |  | < .001 |  |
|  |  | standard fear - neutral |  | -0.09 |  | 0.07 |  | -0.28 |  | 0.10 |  | 560.00 |  | -1.24 |  | 0.216 |  |
|  |  | bad news - neutral |  | -0.54 |  | 0.07 |  | -0.73 |  | -0.36 |  | 560.00 |  | -7.42 |  | < .001 |  |
|  |  | health anxiety [IAS] |  | 0.19 |  | 0.06 |  | 0.02 |  | 0.35 |  | 138.00 |  | 2.88 |  | 0.025 |  |
|  |  | gender |  | 0.11 |  | 0.14 |  | -0.25 |  | 0.48 |  | 138.00 |  | 0.81 |  | 1.000 |  |
|  |  | age |  | 0.00 |  | 0.01 |  | -0.01 |  | 0.02 |  | 138.00 |  | 0.49 |  | 1.000 |  |
|  |  | body symptoms - neutral ✻ health anxiety [IAS] |  | 0.10 |  | 0.07 |  | -0.08 |  | 0.29 |  | 560.00 |  | 1.42 |  | 0.780 |  |
|  |  | signs of severe disease - neutral ✻ health anxiety [IAS] |  | 0.28 |  | 0.07 |  | 0.09 |  | 0.47 |  | 560.00 |  | 3.83 |  | < .001 |  |
|  |  | standard fear - neutral ✻ health anxiety [IAS] |  | -0.05 |  | 0.07 |  | -0.24 |  | 0.14 |  | 560.00 |  | -0.64 |  | 1.000 |  |
|  |  | bad news - neutral ✻ health anxiety [IAS] |  | 0.21 |  | 0.07 |  | 0.02 |  | 0.40 |  | 560.00 |  | 2.90 |  | 0.020 |  |
|  | | | | | | | | | | | | | | | | | |

***Table S6.*** Results of the simple effects of category on anxiety for conditional values of the IAS (5^th^, 50^th^, and 95^th^ percentile of IAS)

|  | | | | | | | | | | | | | | | | | |
| --- | --- | --- | --- | --- | --- | --- | --- | --- | --- | --- | --- | --- | --- | --- | --- | --- | --- |
| **Moderator levels** | |  | | | | | | **99% Confidence Interval** | | | |  | | | | | |
| **health anxiety [IAS]** | | **contrast** | | **Estimate** | | **SE** | | **Lower** | | **Upper** | | **df** | | **t** | | **p** | |
| 5th |  | body symptoms (vs. neutral) - fear (vs. neutral) |  | -1.29 |  | 0.09 |  | -1.52 |  | -1.05 |  | 420.00 |  | -14.03 |  | < .001 |  |
|  |  | signs of severe disease (vs. neutral) - fear (vs. neutral) |  | -1.13 |  | 0.09 |  | -1.37 |  | -0.89 |  | 420.00 |  | -12.34 |  | < .001 |  |
|  |  | bad news (vs. neutral) - fear (vs. neutral) |  | -0.88 |  | 0.09 |  | -1.12 |  | -0.64 |  | 420.00 |  | -9.60 |  | < .001 |  |
| 50th |  | body symptoms (vs. neutral) - fear (vs. neutral) |  | -1.15 |  | 0.06 |  | -1.30 |  | -1.00 |  | 420.00 |  | -19.82 |  | < .001 |  |
|  |  | signs of severe disease (vs. neutral) - fear (vs. neutral) |  | -0.93 |  | 0.06 |  | -1.08 |  | -0.78 |  | 420.00 |  | -16.13 |  | < .001 |  |
|  |  | bad news (vs. neutral) - fear (vs. neutral) |  | -0.68 |  | 0.06 |  | -0.83 |  | -0.53 |  | 420.00 |  | -11.70 |  | < .001 |  |
| 95th |  | body symptoms (vs. neutral) - fear (vs. neutral) |  | -0.85 |  | 0.13 |  | -1.19 |  | -0.52 |  | 420.00 |  | -6.54 |  | < .001 |  |
|  |  | signs of severe disease (vs. neutral) - fear (vs. neutral) |  | -0.52 |  | 0.13 |  | -0.86 |  | -0.18 |  | 420.00 |  | -3.98 |  | < .001 |  |
|  |  | bad news (vs. neutral) - fear (vs. neutral) |  | -0.25 |  | 0.13 |  | -0.59 |  | 0.09 |  | 420.00 |  | -1.93 |  | 0.269 |  |
|  | | | | | | | | | | | | | | | | | |

***Table S7.*** Results of the simple effects of category on displeasure for conditional values of the IAS (5^th^, 50^th^, and 95^th^ percentile of IAS)

|  | | | | | | | | | | | | | | | | | |
| --- | --- | --- | --- | --- | --- | --- | --- | --- | --- | --- | --- | --- | --- | --- | --- | --- | --- |
| **Moderator levels** | |  | | | | | | **99% Confidence Interval** | | | |  | | | | | |
| **health anxiety [IAS]** | | **contrast** | | **Estimate** | | **SE** | | **Lower** | | **Upper** | | **df** | | **t** | | **p** | |
| 5th |  | body symptoms (vs. neutral) - fear (vs. neutral) |  | -0.76 |  | 0.07 |  | -0.96 |  | -0.57 |  | 420.00 |  | -10.23 |  | < .001 |  |
|  |  | signs of severe disease (vs. neutral) - fear (vs. neutral) |  | -0.90 |  | 0.07 |  | -1.09 |  | -0.71 |  | 420.00 |  | -12.09 |  | < .001 |  |
|  |  | bad news (vs. neutral) - fear (vs. neutral) |  | -0.80 |  | 0.07 |  | -1.00 |  | -0.61 |  | 420.00 |  | -10.79 |  | < .001 |  |
| 50th |  | body symptoms (vs. neutral) - fear (vs. neutral) |  | -0.65 |  | 0.05 |  | -0.77 |  | -0.53 |  | 420.00 |  | -13.81 |  | < .001 |  |
|  |  | signs of severe disease (vs. neutral) - fear (vs. neutral) |  | -0.74 |  | 0.05 |  | -0.86 |  | -0.61 |  | 420.00 |  | -15.66 |  | < .001 |  |
|  |  | bad news (vs. neutral) - fear (vs. neutral) |  | -0.61 |  | 0.05 |  | -0.73 |  | -0.49 |  | 420.00 |  | -13.03 |  | < .001 |  |
| 95th |  | body symptoms (vs. neutral) - fear (vs. neutral) |  | -0.41 |  | 0.11 |  | -0.69 |  | -0.14 |  | 420.00 |  | -3.90 |  | < .001 |  |
|  |  | signs of severe disease (vs. neutral) - fear (vs. neutral) |  | -0.39 |  | 0.11 |  | -0.67 |  | -0.12 |  | 420.00 |  | -3.70 |  | < .001 |  |
|  |  | bad news (vs. neutral) - fear (vs. neutral) |  | -0.22 |  | 0.11 |  | -0.49 |  | 0.06 |  | 420.00 |  | -2.03 |  | 0.215 |  |
|  | | | | | | | | | | | | | | | | | |
|  | | | | | | | | | | | | | | | | | |

***Table S8.*** Results of the simple effects of category on arousal for conditional values of the IAS (5^th^, 50^th^, and 95^th^ percentile of IAS)

|  | | | | | | | | | | | | | | | | | |
| --- | --- | --- | --- | --- | --- | --- | --- | --- | --- | --- | --- | --- | --- | --- | --- | --- | --- |
| **Moderator levels** | |  | | | | | | **99% Confidence Interval** | | | |  | | | | | |
| **health anxiety [IAS]** | | **contrast** | | **Estimate** | | **SE** | | **Lower** | | **Upper** | | **df** | | **t** | | **p** | |
| 5th |  | body symptoms (vs. neutral) - fear (vs. neutral) |  | -1.27 |  | 0.09 |  | -1.49 |  | -1.05 |  | 420.00 |  | -14.89 |  | < .001 |  |
|  |  | signs of severe disease (vs. neutral) - fear (vs. neutral) |  | -1.26 |  | 0.09 |  | -1.48 |  | -1.04 |  | 420.00 |  | -14.82 |  | < .001 |  |
|  |  | bad news (vs. neutral) - fear (vs. neutral) |  | -0.93 |  | 0.09 |  | -1.15 |  | -0.71 |  | 420.00 |  | -10.95 |  | < .001 |  |
| 50th |  | body symptoms (vs. neutral) - fear (vs. neutral) |  | -1.11 |  | 0.05 |  | -1.25 |  | -0.97 |  | 420.00 |  | -20.69 |  | < .001 |  |
|  |  | signs of severe disease (vs. neutral) - fear (vs. neutral) |  | -1.04 |  | 0.05 |  | -1.18 |  | -0.90 |  | 420.00 |  | -19.43 |  | < .001 |  |
|  |  | bad news (vs. neutral) - fear (vs. neutral) |  | -0.72 |  | 0.05 |  | -0.86 |  | -0.58 |  | 420.00 |  | -13.44 |  | < .001 |  |
| 95th |  | body symptoms (vs. neutral) - fear (vs. neutral) |  | -0.78 |  | 0.12 |  | -1.10 |  | -0.47 |  | 420.00 |  | -6.48 |  | < .001 |  |
|  |  | signs of severe disease (vs. neutral) - fear (vs. neutral) |  | -0.59 |  | 0.12 |  | -0.90 |  | -0.28 |  | 420.00 |  | -4.87 |  | < .001 |  |
|  |  | bad news (vs. neutral) - fear (vs. neutral) |  | -0.28 |  | 0.12 |  | -0.60 |  | 0.03 |  | 420.00 |  | -2.35 |  | 0.095 |  |
|  | | | | | | | | | | | | | | | | | |
|  | | | | | | | | | | | | | | | | | |

***Table S9.*** Results of the simple effects of category on avoidance tendency for conditional values of the IAS (5^th^, 50^th^, and 95^th^ percentile of IAS)

|  | | | | | | | | | | | | | | | | | |
| --- | --- | --- | --- | --- | --- | --- | --- | --- | --- | --- | --- | --- | --- | --- | --- | --- | --- |
| **Moderator levels** | |  | | | | | | **99% Confidence Interval** | | | |  | | | | | |
| **health anxiety [IAS]** | | **contrast** | | **Estimate** | | **SE** | | **Lower** | | **Upper** | | **df** | | **t** | | **p** | |
| 5th |  | body symptoms (vs. neutral) - fear (vs. neutral) |  | -0.83 |  | 0.09 |  | -1.08 |  | -0.59 |  | 420.00 |  | -8.87 |  | < .001 |  |
|  |  | signs of severe disease (vs. neutral) - fear (vs. neutral) |  | -0.96 |  | 0.09 |  | -1.20 |  | -0.71 |  | 420.00 |  | -10.19 |  | < .001 |  |
|  |  | bad news (vs. neutral) - fear (vs. neutral) |  | -0.96 |  | 0.09 |  | -1.20 |  | -0.72 |  | 420.00 |  | -10.23 |  | < .001 |  |
| 50th |  | body symptoms (vs. neutral) - fear (vs. neutral) |  | -0.71 |  | 0.06 |  | -0.86 |  | -0.56 |  | 420.00 |  | -12.02 |  | < .001 |  |
|  |  | signs of severe disease (vs. neutral) - fear (vs. neutral) |  | -0.75 |  | 0.06 |  | -0.90 |  | -0.60 |  | 420.00 |  | -12.69 |  | < .001 |  |
|  |  | bad news (vs. neutral) - fear (vs. neutral) |  | -0.73 |  | 0.06 |  | -0.88 |  | -0.57 |  | 420.00 |  | -12.30 |  | < .001 |  |
| 95th |  | body symptoms (vs. neutral) - fear (vs. neutral) |  | -0.46 |  | 0.13 |  | -0.81 |  | -0.11 |  | 420.00 |  | -3.44 |  | < .001 |  |
|  |  | signs of severe disease (vs. neutral) - fear (vs. neutral) |  | -0.32 |  | 0.13 |  | -0.67 |  | 0.02 |  | 420.00 |  | -2.43 |  | 0.08 |  |
|  |  | bad news (vs. neutral) - fear (vs. neutral) |  | -0.25 |  | 0.13 |  | -0.59 |  | 0.10 |  | 420.00 |  | -1.84 |  | 0.330 |  |
|  | | | | | | | | | | | | | | | | | |
|  | | | | | | | | | | | | | | | | | |

***Table S10.*** Results of the simple effects of category on vividness for conditional values of the IAS (5^th^, 50^th^, and 95^th^ percentile of IAS)

|  | | | | | | | | | | | | | | | | | |
| --- | --- | --- | --- | --- | --- | --- | --- | --- | --- | --- | --- | --- | --- | --- | --- | --- | --- |
| **Moderator levels** | |  | | | | | | **99% Confidence Interval** | | | |  | | | | | |
| **health anxiety [IAS]** | | **contrast** | | **Estimate** | | **SE** | | **Lower** | | **Upper** | | **df** | | **t** | | **p** | |
| 5th |  | body symptoms (vs. neutral) - fear (vs. neutral) |  | -0.51 |  | 0.11 |  | -0.78 |  | -0.23 |  | 420.00 |  | -4.75 |  | < .001 |  |
|  |  | signs of severe disease (vs. neutral) - fear (vs. neutral) |  | -1.14 |  | 0.11 |  | -1.41 |  | -0.86 |  | 420.00 |  | -10.69 |  | < .001 |  |
|  |  | bad news (vs. neutral) - fear (vs. neutral) |  | -0.77 |  | 0.11 |  | -1.04 |  | -0.49 |  | 420.00 |  | -7.21 |  | < .001 |  |
| 50th |  | body symptoms (vs. neutral) - fear (vs. neutral) |  | -0.34 |  | 0.07 |  | -0.52 |  | -0.17 |  | 420.00 |  | -5.14 |  | < .001 |  |
|  |  | signs of severe disease (vs. neutral) - fear (vs. neutral) |  | -0.79 |  | 0.07 |  | -0.96 |  | -0.62 |  | 420.00 |  | -11.77 |  | < .001 |  |
|  |  | bad news (vs. neutral) - fear (vs. neutral) |  | -0.49 |  | 0.07 |  | -0.66 |  | -0.32 |  | 420.00 |  | -7.33 |  | < .001 |  |
| 95th |  | body symptoms (vs. neutral) - fear (vs. neutral) |  | -0.01 |  | 0.15 |  | -0.40 |  | 0.38 |  | 420.00 |  | -0.07 |  | 1.000 |  |
|  |  | signs of severe disease (vs. neutral) - fear (vs. neutral) |  | -0.07 |  | 0.15 |  | -0.46 |  | 0.32 |  | 420.00 |  | -0.44 |  | 1.000 |  |
|  |  | bad news (vs. neutral) - fear (vs. neutral) |  | 0.08 |  | 0.15 |  | -0.31 |  | 0.47 |  | 420.00 |  | 0.53 |  | 1.000 |  |
|  | | | | | | | | | | | | | | | | | |
|  | | | | | | | | | | | | | | | | | |

***Table S11.*** Parameter estimates from mixed effects regression models examining the impact of mental imagery content, health anxiety, anxiety sensitivity, depressive symptoms, anxiety symptoms, trait anxiety, and somatic symptom severity on reported anxiety.

|  | | | | | | | | | | | | | | | | | |
| --- | --- | --- | --- | --- | --- | --- | --- | --- | --- | --- | --- | --- | --- | --- | --- | --- | --- |
|  | | | | | | | | **99% Confidence Interval** | | | |  | | | | | |
|  | | **Effect** | | **Estimate** | | **SE** | | **Lower** | | **Upper** | | **df** | | **t** | | **p** | |
|  |  | (Intercept) |  | -0.00 |  | 0.04 |  | -0.11 |  | 0.11 |  | 133.00 |  | -0.00 |  | 1.000 |  |
|  |  | body symptoms - neutral |  | 1.19 |  | 0.06 |  | 1.05 |  | 1.34 |  | 540.00 |  | 21.18 |  | < .001 |  |
|  |  | signs of severe disease - neutral |  | 1.39 |  | 0.06 |  | 1.24 |  | 1.53 |  | 540.00 |  | 24.66 |  | < .001 |  |
|  |  | standard fear - neutral |  | 2.18 |  | 0.06 |  | 2.04 |  | 2.33 |  | 540.00 |  | 38.72 |  | < .001 |  |
|  |  | bad news - neutral |  | 1.62 |  | 0.06 |  | 1.47 |  | 1.76 |  | 540.00 |  | 28.68 |  | < .001 |  |
|  |  | health anxiety [IAS] |  | 0.17 |  | 0.06 |  | 0.02 |  | 0.31 |  | 133.00 |  | 2.99 |  | 0.015 |  |
|  |  | age |  | -0.07 |  | 0.04 |  | -0.18 |  | 0.04 |  | 133.00 |  | -1.62 |  | 0.535 |  |
|  |  | gender |  | 0.10 |  | 0.04 |  | -0.01 |  | 0.22 |  | 133.00 |  | 2.31 |  | 0.115 |  |
|  |  | anxiety sensitivity [ASI-3] |  | 0.13 |  | 0.06 |  | -0.03 |  | 0.29 |  | 133.00 |  | 2.16 |  | 0.165 |  |
|  |  | somatic symptom severity [PHQ-15] |  | -0.11 |  | 0.06 |  | -0.26 |  | 0.04 |  | 133.00 |  | -1.90 |  | 0.325 |  |
|  |  | depressive symptoms [PHQ-2] |  | -0.03 |  | 0.05 |  | -0.17 |  | 0.11 |  | 133.00 |  | -0.63 |  | 1.000 |  |
|  |  | anxiety symptoms [GAD-2] |  | 0.00 |  | 0.06 |  | -0.15 |  | 0.16 |  | 133.00 |  | 0.05 |  | 1.000 |  |
|  |  | trait anxiety [STAI] |  | 0.02 |  | 0.06 |  | -0.14 |  | 0.17 |  | 133.00 |  | 0.27 |  | 1.000 |  |
|  |  | body symptoms - neutral ✻ health anxiety [IAS] |  | 0.25 |  | 0.08 |  | 0.05 |  | 0.44 |  | 540.00 |  | 3.21 |  | 0.001 |  |
|  |  | signs of severe disease - neutral ✻ health anxiety [IAS] |  | 0.27 |  | 0.08 |  | 0.07 |  | 0.47 |  | 540.00 |  | 3.49 |  | < .001 |  |
|  |  | standard fear - neutral ✻ health anxiety [IAS] |  | 0.18 |  | 0.08 |  | -0.02 |  | 0.38 |  | 540.00 |  | 2.35 |  | 0.019 |  |
|  |  | bad news - neutral ✻ health anxiety [IAS] |  | 0.33 |  | 0.08 |  | 0.14 |  | 0.53 |  | 540.00 |  | 4.36 |  | < .001 |  |
|  |  | body symptoms - neutral ✻ anxiety sensitivity [ASI-3] |  | 0.06 |  | 0.08 |  | -0.16 |  | 0.27 |  | 540.00 |  | 0.70 |  | 1.000 |  |
|  |  | signs of severe disease - neutral ✻ anxiety sensitivity [ASI-3] |  | 0.07 |  | 0.08 |  | -0.14 |  | 0.29 |  | 540.00 |  | 0.86 |  | 1.000 |  |
|  |  | standard fear - neutral ✻ anxiety sensitivity [ASI-3] |  | 0.01 |  | 0.08 |  | -0.21 |  | 0.22 |  | 540.00 |  | 0.06 |  | 1.000 |  |
|  |  | bad news - neutral ✻ anxiety sensitivity [ASI-3] |  | 0.04 |  | 0.08 |  | -0.17 |  | 0.26 |  | 540.00 |  | 0.49 |  | 1.000 |  |
|  |  | body symptoms - neutral ✻ somatic symptom severity [PHQ-15] |  | -0.09 |  | 0.08 |  | -0.29 |  | 0.12 |  | 540.00 |  | -1.09 |  | 1.000 |  |
|  |  | signs of severe disease - neutral ✻ somatic symptom severity [PHQ-15] |  | -0.05 |  | 0.08 |  | -0.25 |  | 0.15 |  | 540.00 |  | -0.66 |  | 1.000 |  |
|  |  | standard fear - neutral ✻ somatic symptom severity [PHQ-15] |  | -0.06 |  | 0.08 |  | -0.26 |  | 0.14 |  | 540.00 |  | -0.76 |  | 1.000 |  |
|  |  | bad news - neutral ✻ somatic symptom severity [PHQ-15] |  | -0.02 |  | 0.08 |  | -0.22 |  | 0.19 |  | 540.00 |  | -0.20 |  | 1.000 |  |
|  |  | body symptoms - neutral ✻ depressive symptoms [PHQ-2] |  | -0.14 |  | 0.07 |  | -0.33 |  | 0.05 |  | 540.00 |  | -1.93 |  | 0.270 |  |
|  |  | signs of severe disease - neutral ✻ depressive symptoms [PHQ-2] |  | -0.17 |  | 0.07 |  | -0.35 |  | 0.02 |  | 540.00 |  | -2.30 |  | 0.110 |  |
|  |  | standard fear - neutral ✻ depressive symptoms [PHQ-2] |  | -0.19 |  | 0.07 |  | -0.38 |  | -0.00 |  | 540.00 |  | -2.62 |  | 0.045 |  |
|  |  | bad news - neutral ✻ depressive symptoms [PHQ-2] |  | -0.10 |  | 0.07 |  | -0.29 |  | 0.08 |  | 540.00 |  | -1.44 |  | 0.755 |  |
|  |  | body symptoms - neutral ✻ anxiety symptoms [GAD-2] |  | 0.07 |  | 0.08 |  | -0.14 |  | 0.27 |  | 540.00 |  | 0.81 |  | 1.000 |  |
|  |  | signs of severe disease - neutral ✻ anxiety symptoms [GAD-2] |  | 0.02 |  | 0.08 |  | -0.19 |  | 0.23 |  | 540.00 |  | 0.26 |  | 1.000 |  |
|  |  | standard fear - neutral ✻ anxiety symptoms [GAD-2] |  | 0.02 |  | 0.08 |  | -0.18 |  | 0.23 |  | 540.00 |  | 0.30 |  | 1.000 |  |
|  |  | bad news - neutral ✻ anxiety symptoms [GAD-2] |  | -0.03 |  | 0.08 |  | -0.23 |  | 0.18 |  | 540.00 |  | -0.31 |  | 1.000 |  |
|  |  | body symptoms - neutral ✻ trait anxiety [STAI] |  | 0.01 |  | 0.08 |  | -0.19 |  | 0.22 |  | 540.00 |  | 0.16 |  | 1.000 |  |
|  |  | signs of severe disease - neutral ✻ trait anxiety [STAI] |  | 0.06 |  | 0.08 |  | -0.14 |  | 0.27 |  | 540.00 |  | 0.78 |  | 1.000 |  |
|  |  | standard fear - neutral ✻ trait anxiety [STAI] |  | 0.02 |  | 0.08 |  | -0.19 |  | 0.22 |  | 540.00 |  | 0.21 |  | 1.000 |  |
|  |  | bad news - neutral ✻ trait anxiety [STAI] |  | -0.06 |  | 0.08 |  | -0.26 |  | 0.15 |  | 540.00 |  | -0.72 |  | 1.000 |  |
|  | | | | | | | | | | | | | | | | | |

***Table S12.*** Parameter estimates from mixed effects regression models examining the impact of mental imagery content, health anxiety, anxiety sensitivity, depressive symptoms, anxiety symptoms, trait anxiety, and somatic symptom severity on reported displeasure.

|  | | | | | | | | | | | | | | | | | |
| --- | --- | --- | --- | --- | --- | --- | --- | --- | --- | --- | --- | --- | --- | --- | --- | --- | --- |
|  | | | | | | | | **99% Confidence Interval** | | | |  | | | | | |
|  | | **Effect** | | **Estimate** | | **SE** | | **Lower** | | **Upper** | | **df** | | **t** | | **p** | |
|  |  | (Intercept) |  | 0.00 |  | 0.03 |  | -0.07 |  | 0.07 |  | 133.00 |  | 0.00 |  | 1.000 |  |
|  |  | body symptoms - neutral |  | 1.94 |  | 0.05 |  | 1.81 |  | 2.07 |  | 540.00 |  | 38.28 |  | < .001 |  |
|  |  | signs of severe disease - neutral |  | 1.88 |  | 0.05 |  | 1.75 |  | 2.01 |  | 540.00 |  | 37.00 |  | < .001 |  |
|  |  | standard fear - neutral |  | 2.46 |  | 0.05 |  | 2.33 |  | 2.60 |  | 540.00 |  | 48.60 |  | < .001 |  |
|  |  | bad news - neutral |  | 1.98 |  | 0.05 |  | 1.85 |  | 2.11 |  | 540.00 |  | 39.09 |  | < .001 |  |
|  |  | health anxiety [IAS] |  | 0.10 |  | 0.04 |  | 0.00 |  | 0.19 |  | 133.00 |  | 2.69 |  | 0.040 |  |
|  |  | age |  | -0.07 |  | 0.03 |  | -0.15 |  | -0.00 |  | 133.00 |  | -2.67 |  | 0.040 |  |
|  |  | anxiety sensitivity [ASI-3] |  | 0.03 |  | 0.04 |  | -0.07 |  | 0.13 |  | 133.00 |  | 0.71 |  | 1.000 |  |
|  |  | somatic symptom severity [PHQ-15] |  | -0.13 |  | 0.04 |  | -0.22 |  | -0.03 |  | 133.00 |  | -3.30 |  | 0.005 |  |
|  |  | depressive symptoms [PHQ-2] |  | -0.02 |  | 0.03 |  | -0.11 |  | 0.07 |  | 133.00 |  | -0.54 |  | 1.000 |  |
|  |  | anxiety symptoms [GAD-2] |  | 0.06 |  | 0.04 |  | -0.04 |  | 0.16 |  | 133.00 |  | 1.47 |  | 0.720 |  |
|  |  | gender |  | 0.12 |  | 0.06 |  | -0.04 |  | 0.29 |  | 133.00 |  | 1.94 |  | 0.270 |  |
|  |  | trait anxiety [STAI] |  | 0.03 |  | 0.04 |  | -0.07 |  | 0.13 |  | 133.00 |  | 0.76 |  | 1.000 |  |
|  |  | body symptoms - neutral ✻ health anxiety [IAS] |  | 0.19 |  | 0.07 |  | 0.01 |  | 0.37 |  | 540.00 |  | 2.76 |  | 0.030 |  |
|  |  | signs of severe disease - neutral ✻ health anxiety [IAS] |  | 0.22 |  | 0.07 |  | 0.04 |  | 0.39 |  | 540.00 |  | 3.13 |  | 0.010 |  |
|  |  | standard fear - neutral ✻ health anxiety [IAS] |  | 0.18 |  | 0.07 |  | -0.00 |  | 0.35 |  | 540.00 |  | 2.55 |  | 0.055 |  |
|  |  | bad news - neutral ✻ health anxiety [IAS] |  | 0.30 |  | 0.07 |  | 0.13 |  | 0.48 |  | 540.00 |  | 4.39 |  | < .001 |  |
|  |  | body symptoms - neutral ✻ anxiety sensitivity [ASI-3] |  | -0.01 |  | 0.08 |  | -0.21 |  | 0.18 |  | 540.00 |  | -0.18 |  | 1.000 |  |
|  |  | signs of severe disease - neutral ✻ anxiety sensitivity [ASI-3] |  | 0.01 |  | 0.08 |  | -0.18 |  | 0.20 |  | 540.00 |  | 0.13 |  | 1.000 |  |
|  |  | standard fear - neutral ✻ anxiety sensitivity [ASI-3] |  | -0.08 |  | 0.08 |  | -0.27 |  | 0.12 |  | 540.00 |  | -1.02 |  | 1.000 |  |
|  |  | bad news - neutral ✻ anxiety sensitivity [ASI-3] |  | -0.08 |  | 0.08 |  | -0.27 |  | 0.12 |  | 540.00 |  | -1.00 |  | 1.000 |  |
|  |  | body symptoms - neutral ✻ somatic symptom severity [PHQ-15] |  | -0.13 |  | 0.07 |  | -0.31 |  | 0.05 |  | 540.00 |  | -1.85 |  | 0.325 |  |
|  |  | signs of severe disease - neutral ✻ somatic symptom severity [PHQ-15] |  | -0.15 |  | 0.07 |  | -0.33 |  | 0.03 |  | 540.00 |  | -2.10 |  | 0.180 |  |
|  |  | standard fear - neutral ✻ somatic symptom severity [PHQ-15] |  | -0.09 |  | 0.07 |  | -0.27 |  | 0.09 |  | 540.00 |  | -1.26 |  | 1.000 |  |
|  |  | bad news - neutral ✻ somatic symptom severity [PHQ-15] |  | -0.12 |  | 0.07 |  | -0.31 |  | 0.06 |  | 540.00 |  | -1.76 |  | 0.395 |  |
|  |  | body symptoms - neutral ✻ depressive symptoms [PHQ-2] |  | -0.13 |  | 0.07 |  | -0.30 |  | 0.04 |  | 540.00 |  | -1.97 |  | 0.250 |  |
|  |  | signs of severe disease - neutral ✻ depressive symptoms [PHQ-2] |  | -0.15 |  | 0.07 |  | -0.32 |  | 0.02 |  | 540.00 |  | -2.28 |  | 0.115 |  |
|  |  | standard fear - neutral ✻ depressive symptoms [PHQ-2] |  | -0.15 |  | 0.07 |  | -0.32 |  | 0.02 |  | 540.00 |  | -2.31 |  | 0.105 |  |
|  |  | bad news - neutral ✻ depressive symptoms [PHQ-2] |  | -0.13 |  | 0.07 |  | -0.30 |  | 0.03 |  | 540.00 |  | -2.06 |  | 0.200 |  |
|  |  | body symptoms - neutral ✻ anxiety symptoms [GAD-2] |  | -0.04 |  | 0.07 |  | -0.23 |  | 0.14 |  | 540.00 |  | -0.60 |  | 1.000 |  |
|  |  | signs of severe disease - neutral ✻ anxiety symptoms [GAD-2] |  | 0.00 |  | 0.07 |  | -0.18 |  | 0.19 |  | 540.00 |  | 0.06 |  | 1.000 |  |
|  |  | standard fear - neutral ✻ anxiety symptoms [GAD-2] |  | -0.07 |  | 0.07 |  | -0.26 |  | 0.12 |  | 540.00 |  | -0.97 |  | 1.000 |  |
|  |  | bad news - neutral ✻ anxiety symptoms [GAD-2] |  | 0.05 |  | 0.07 |  | -0.14 |  | 0.24 |  | 540.00 |  | 0.70 |  | 1.000 |  |
|  |  | body symptoms - neutral ✻ trait anxiety [STAI] |  | 0.12 |  | 0.07 |  | -0.06 |  | 0.31 |  | 540.00 |  | 1.69 |  | 0.455 |  |
|  |  | signs of severe disease - neutral ✻ trait anxiety [STAI] |  | 0.11 |  | 0.07 |  | -0.07 |  | 0.30 |  | 540.00 |  | 1.57 |  | 0.590 |  |
|  |  | standard fear - neutral ✻ trait anxiety [STAI] |  | 0.05 |  | 0.07 |  | -0.14 |  | 0.23 |  | 540.00 |  | 0.65 |  | 1.000 |  |
|  |  | bad news - neutral ✻ trait anxiety [STAI] |  | 0.02 |  | 0.07 |  | -0.17 |  | 0.20 |  | 540.00 |  | 0.24 |  | 1.000 |  |
|  | | | | | | | | | | | | | | | | | |

***Table S13.*** Parameter estimates from mixed effects regression models examining the impact of mental imagery content, health anxiety, anxiety sensitivity, depressive symptoms, anxiety symptoms, trait anxiety, and somatic symptom severity on reported arousal.

|  | | | | | | | | | | | | | | | | | |
| --- | --- | --- | --- | --- | --- | --- | --- | --- | --- | --- | --- | --- | --- | --- | --- | --- | --- |
|  | | | | | | | | **99% Confidence Interval** | | | |  | | | | | |
|  | | **Effect** | | **Estimate** | | **SE** | | **Lower** | | **Upper** | | **df** | | **t** | | **p** | |
|  |  | (Intercept) |  | -0.00 |  | 0.03 |  | -0.09 |  | 0.09 |  | 133.00 |  | -0.00 |  | 1.000 |  |
|  |  | body symptoms - neutral |  | 1.57 |  | 0.05 |  | 1.44 |  | 1.69 |  | 540.00 |  | 32.39 |  | < .001 |  |
|  |  | signs of severe disease - neutral |  | 1.63 |  | 0.05 |  | 1.51 |  | 1.76 |  | 540.00 |  | 33.65 |  | < .001 |  |
|  |  | standard fear - neutral |  | 2.42 |  | 0.05 |  | 2.30 |  | 2.55 |  | 540.00 |  | 50.04 |  | < .001 |  |
|  |  | bad news - neutral |  | 1.88 |  | 0.05 |  | 1.76 |  | 2.01 |  | 540.00 |  | 38.87 |  | < .001 |  |
|  |  | health anxiety [IAS] |  | 0.10 |  | 0.05 |  | -0.02 |  | 0.23 |  | 133.00 |  | 2.17 |  | 0.160 |  |
|  |  | age |  | -0.11 |  | 0.04 |  | -0.20 |  | -0.02 |  | 133.00 |  | -3.05 |  | 0.015 |  |
|  |  | gender |  | 0.08 |  | 0.04 |  | -0.01 |  | 0.18 |  | 133.00 |  | 2.19 |  | 0.150 |  |
|  |  | anxiety sensitivity [ASI-3] |  | 0.10 |  | 0.05 |  | -0.04 |  | 0.23 |  | 133.00 |  | 1.89 |  | 0.305 |  |
|  |  | somatic symptom severity [PHQ-15] |  | -0.13 |  | 0.05 |  | -0.26 |  | -0.00 |  | 133.00 |  | -2.64 |  | 0.045 |  |
|  |  | depressive symptoms [PHQ-2] |  | -0.03 |  | 0.05 |  | -0.14 |  | 0.09 |  | 133.00 |  | -0.59 |  | 1.000 |  |
|  |  | anxiety symptoms [GAD-2] |  | 0.04 |  | 0.05 |  | -0.09 |  | 0.16 |  | 133.00 |  | 0.70 |  | 1.000 |  |
|  |  | trait anxiety [STAI] |  | 0.00 |  | 0.01 |  | -0.02 |  | 0.03 |  | 133.00 |  | 0.37 |  | 1.000 |  |
|  |  | body symptoms - neutral ✻ health anxiety [IAS] |  | 0.22 |  | 0.07 |  | 0.05 |  | 0.39 |  | 540.00 |  | 3.31 |  | < .001 |  |
|  |  | signs of severe disease - neutral ✻ health anxiety [IAS] |  | 0.20 |  | 0.07 |  | 0.03 |  | 0.37 |  | 540.00 |  | 3.04 |  | 0.010 |  |
|  |  | standard fear - neutral ✻ health anxiety [IAS] |  | 0.14 |  | 0.07 |  | -0.03 |  | 0.31 |  | 540.00 |  | 2.19 |  | 0.145 |  |
|  |  | bad news - neutral ✻ health anxiety [IAS] |  | 0.28 |  | 0.07 |  | 0.11 |  | 0.45 |  | 540.00 |  | 4.23 |  | < .001 |  |
|  |  | body symptoms - neutral ✻ anxiety sensitivity [ASI-3] |  | 0.03 |  | 0.07 |  | -0.15 |  | 0.22 |  | 540.00 |  | 0.46 |  | 1.000 |  |
|  |  | signs of severe disease - neutral ✻ anxiety sensitivity [ASI-3] |  | 0.07 |  | 0.07 |  | -0.12 |  | 0.25 |  | 540.00 |  | 0.91 |  | 1.000 |  |
|  |  | standard fear - neutral ✻ anxiety sensitivity [ASI-3] |  | -0.11 |  | 0.07 |  | -0.29 |  | 0.08 |  | 540.00 |  | -1.51 |  | 0.660 |  |
|  |  | bad news - neutral ✻ anxiety sensitivity [ASI-3] |  | -0.03 |  | 0.07 |  | -0.22 |  | 0.15 |  | 540.00 |  | -0.43 |  | 1.000 |  |
|  |  | body symptoms - neutral ✻ somatic symptom severity [PHQ-15] |  | -0.21 |  | 0.07 |  | -0.38 |  | -0.03 |  | 540.00 |  | -3.06 |  | 0.010 |  |
|  |  | signs of severe disease - neutral ✻ somatic symptom severity [PHQ-15] |  | -0.18 |  | 0.07 |  | -0.36 |  | -0.01 |  | 540.00 |  | -2.73 |  | 0.175 |  |
|  |  | standard fear - neutral ✻ somatic symptom severity [PHQ-15] |  | -0.12 |  | 0.07 |  | -0.30 |  | 0.05 |  | 540.00 |  | -1.85 |  | 0.325 |  |
|  |  | bad news - neutral ✻ somatic symptom severity [PHQ-15] |  | -0.18 |  | 0.07 |  | -0.35 |  | -0.01 |  | 540.00 |  | -2.69 |  | 0.035 |  |
|  |  | body symptoms - neutral ✻ depressive symptoms [PHQ-2] |  | -0.02 |  | 0.06 |  | -0.18 |  | 0.14 |  | 540.00 |  | -0.38 |  | 1.000 |  |
|  |  | signs of severe disease - neutral ✻ depressive symptoms [PHQ-2] |  | -0.09 |  | 0.06 |  | -0.25 |  | 0.07 |  | 540.00 |  | -1.47 |  | 0.710 |  |
|  |  | standard fear - neutral ✻ depressive symptoms [PHQ-2] |  | -0.05 |  | 0.06 |  | -0.21 |  | 0.11 |  | 540.00 |  | -0.75 |  | 1.000 |  |
|  |  | bad news - neutral ✻ depressive symptoms [PHQ-2] |  | -0.10 |  | 0.06 |  | -0.26 |  | 0.06 |  | 540.00 |  | -1.64 |  | 0.505 |  |
|  |  | body symptoms - neutral ✻ anxiety symptoms [GAD-2] |  | -0.11 |  | 0.07 |  | -0.29 |  | 0.07 |  | 540.00 |  | -1.58 |  | 0.570 |  |
|  |  | signs of severe disease - neutral ✻ anxiety symptoms [GAD-2] |  | -0.05 |  | 0.07 |  | -0.23 |  | 0.13 |  | 540.00 |  | -0.76 |  | 1.000 |  |
|  |  | standard fear - neutral ✻ anxiety symptoms [GAD-2] |  | -0.14 |  | 0.07 |  | -0.32 |  | 0.04 |  | 540.00 |  | -1.99 |  | 0.235 |  |
|  |  | bad news - neutral ✻ anxiety symptoms [GAD-2] |  | -0.03 |  | 0.07 |  | -0.21 |  | 0.15 |  | 540.00 |  | -0.40 |  | 1.000 |  |
|  |  | body symptoms - neutral ✻ trait anxiety [STAI] |  | 0.02 |  | 0.01 |  | -0.01 |  | 0.05 |  | 540.00 |  | 1.36 |  | 0.875 |  |
|  |  | signs of severe disease - neutral ✻ trait anxiety [STAI] |  | 0.03 |  | 0.01 |  | -0.01 |  | 0.06 |  | 540.00 |  | 2.10 |  | 0.180 |  |
|  |  | standard fear - neutral ✻ trait anxiety [STAI] |  | 0.02 |  | 0.01 |  | -0.01 |  | 0.05 |  | 540.00 |  | 1.98 |  | 0.240 |  |
|  |  | bad news - neutral ✻ trait anxiety [STAI] |  | 0.01 |  | 0.01 |  | -0.02 |  | 0.05 |  | 540.00 |  | 1.20 |  | 1.000 |  |
|  | | | | | | | | | | | | | | | | | |

***Table S14.*** Parameter estimates from mixed effects regression models examining the impact of mental imagery content, health anxiety, anxiety sensitivity, depressive symptoms, anxiety symptoms, trait anxiety, and somatic symptom severity on reported avoidance.

|  | | | | | | | | | | | | | | | | | |
| --- | --- | --- | --- | --- | --- | --- | --- | --- | --- | --- | --- | --- | --- | --- | --- | --- | --- |
|  | | | | | | | | **99% Confidence Interval** | | | |  | | | | | |
|  | | **Effect** | | **Estimate** | | **SE** | | **Lower** | | **Upper** | | **df** | | **t** | | **p** | |
|  |  | (Intercept) |  | -0.00 |  | 0.05 |  | -0.13 |  | 0.13 |  | 133.00 |  | -0.00 |  | 1.000 |  |
|  |  | body symptoms - neutral |  | 1.44 |  | 0.05 |  | 1.30 |  | 1.58 |  | 540.00 |  | 26.40 |  | < .001 |  |
|  |  | signs of severe disease - neutral |  | 1.42 |  | 0.05 |  | 1.28 |  | 1.56 |  | 540.00 |  | 26.01 |  | < .001 |  |
|  |  | standard fear - neutral |  | 2.02 |  | 0.05 |  | 1.88 |  | 2.16 |  | 540.00 |  | 36.96 |  | < .001 |  |
|  |  | bad news - neutral |  | 1.56 |  | 0.05 |  | 1.42 |  | 1.71 |  | 540.00 |  | 28.63 |  | < .001 |  |
|  |  | health anxiety [IAS] |  | 0.14 |  | 0.07 |  | -0.03 |  | 0.31 |  | 133.00 |  | 2.12 |  | 0.180 |  |
|  |  | age |  | -0.04 |  | 0.05 |  | -0.17 |  | 0.10 |  | 133.00 |  | -0.69 |  | 1.000 |  |
|  |  | gender |  | 0.07 |  | 0.05 |  | -0.07 |  | 0.21 |  | 133.00 |  | 1.32 |  | 0.940 |  |
|  |  | anxiety sensitivity [ASI-3] |  | 0.11 |  | 0.07 |  | -0.08 |  | 0.30 |  | 133.00 |  | 1.47 |  | 0.725 |  |
|  |  | somatic symptom severity [PHQ-15] |  | -0.07 |  | 0.07 |  | -0.25 |  | 0.11 |  | 133.00 |  | -1.06 |  | 1.000 |  |
|  |  | depressive symptoms [PHQ-2] |  | 0.00 |  | 0.06 |  | -0.16 |  | 0.17 |  | 133.00 |  | 0.01 |  | 1.000 |  |
|  |  | anxiety symptoms [GAD-2] |  | 0.01 |  | 0.07 |  | -0.17 |  | 0.20 |  | 133.00 |  | 0.19 |  | 1.000 |  |
|  |  | trait anxiety [STAI] |  | -0.00 |  | 0.01 |  | -0.04 |  | 0.03 |  | 133.00 |  | -0.31 |  | 1.000 |  |
|  |  | body symptoms - neutral ✻ health anxiety [IAS] |  | 0.22 |  | 0.07 |  | 0.03 |  | 0.41 |  | 540.00 |  | 2.99 |  | 0.015 |  |
|  |  | signs of severe disease - neutral ✻ health anxiety [IAS] |  | 0.25 |  | 0.07 |  | 0.05 |  | 0.44 |  | 540.00 |  | 3.30 |  | 0.005 |  |
|  |  | standard fear - neutral ✻ health anxiety [IAS] |  | 0.11 |  | 0.07 |  | -0.09 |  | 0.30 |  | 540.00 |  | 1.43 |  | 0.760 |  |
|  |  | bad news - neutral ✻ health anxiety [IAS] |  | 0.28 |  | 0.07 |  | 0.09 |  | 0.47 |  | 540.00 |  | 3.77 |  | < .001 |  |
|  |  | body symptoms - neutral ✻ anxiety sensitivity [ASI-3] |  | -0.05 |  | 0.08 |  | -0.26 |  | 0.16 |  | 540.00 |  | -0.62 |  | 1.000 |  |
|  |  | signs of severe disease - neutral ✻ anxiety sensitivity [ASI-3] |  | 0.03 |  | 0.08 |  | -0.18 |  | 0.24 |  | 540.00 |  | 0.41 |  | 1.000 |  |
|  |  | standard fear - neutral ✻ anxiety sensitivity [ASI-3] |  | 0.01 |  | 0.08 |  | -0.20 |  | 0.22 |  | 540.00 |  | 0.12 |  | 1.000 |  |
|  |  | bad news - neutral ✻ anxiety sensitivity [ASI-3] |  | 0.03 |  | 0.08 |  | -0.18 |  | 0.24 |  | 540.00 |  | 0.34 |  | 1.000 |  |
|  |  | body symptoms - neutral ✻ somatic symptom severity [PHQ-15] |  | -0.06 |  | 0.08 |  | -0.25 |  | 0.14 |  | 540.00 |  | -0.73 |  | 1.000 |  |
|  |  | signs of severe disease - neutral ✻ somatic symptom severity [PHQ-15] |  | -0.07 |  | 0.08 |  | -0.27 |  | 0.12 |  | 540.00 |  | -0.97 |  | 1.000 |  |
|  |  | standard fear - neutral ✻ somatic symptom severity [PHQ-15] |  | 0.01 |  | 0.08 |  | -0.19 |  | 0.20 |  | 540.00 |  | 0.09 |  | 1.000 |  |
|  |  | bad news - neutral ✻ somatic symptom severity [PHQ-15] |  | -0.05 |  | 0.08 |  | -0.25 |  | 0.14 |  | 540.00 |  | -0.71 |  | 1.000 |  |
|  |  | body symptoms - neutral ✻ depressive symptoms [PHQ-2] |  | -0.14 |  | 0.07 |  | -0.32 |  | 0.04 |  | 540.00 |  | -1.98 |  | 0.245 |  |
|  |  | signs of severe disease - neutral ✻ depressive symptoms [PHQ-2] |  | -0.09 |  | 0.07 |  | -0.27 |  | 0.09 |  | 540.00 |  | -1.30 |  | 0.975 |  |
|  |  | standard fear - neutral ✻ depressive symptoms [PHQ-2] |  | -0.17 |  | 0.07 |  | -0.35 |  | 0.01 |  | 540.00 |  | -2.44 |  | 0.075 |  |
|  |  | bad news - neutral ✻ depressive symptoms [PHQ-2] |  | -0.09 |  | 0.07 |  | -0.27 |  | 0.09 |  | 540.00 |  | -1.31 |  | 0.950 |  |
|  |  | body symptoms - neutral ✻ anxiety symptoms [GAD-2] |  | 0.00 |  | 0.08 |  | -0.20 |  | 0.21 |  | 540.00 |  | 0.03 |  | 1.000 |  |
|  |  | signs of severe disease - neutral ✻ anxiety symptoms [GAD-2] |  | 0.01 |  | 0.08 |  | -0.20 |  | 0.21 |  | 540.00 |  | 0.08 |  | 1.000 |  |
|  |  | standard fear - neutral ✻ anxiety symptoms [GAD-2] |  | -0.00 |  | 0.08 |  | -0.20 |  | 0.20 |  | 540.00 |  | -0.01 |  | 1.000 |  |
|  |  | bad news - neutral ✻ anxiety symptoms [GAD-2] |  | -0.02 |  | 0.08 |  | -0.22 |  | 0.18 |  | 540.00 |  | -0.23 |  | 1.000 |  |
|  |  | body symptoms - neutral ✻ trait anxiety [STAI] |  | 0.01 |  | 0.01 |  | -0.03 |  | 0.04 |  | 540.00 |  | 0.63 |  | 1.000 |  |
|  |  | signs of severe disease - neutral ✻ trait anxiety [STAI] |  | 0.00 |  | 0.01 |  | -0.03 |  | 0.04 |  | 540.00 |  | 0.25 |  | 1.000 |  |
|  |  | standard fear - neutral ✻ trait anxiety [STAI] |  | -0.00 |  | 0.01 |  | -0.04 |  | 0.03 |  | 540.00 |  | -0.32 |  | 1.000 |  |
|  |  | bad news - neutral ✻ trait anxiety [STAI] |  | -0.00 |  | 0.01 |  | -0.04 |  | 0.03 |  | 540.00 |  | -0.05 |  | 1.000 |  |
|  | | | | | | | | | | | | | | | | | |

***Table S15.*** Parameter estimates from mixed effects regression models examining the impact of mental imagery content, health anxiety, anxiety sensitivity, depressive symptoms, anxiety symptoms, trait anxiety, and somatic symptom severity on reported vividness.

|  | | | | | | | | | | | | | | | | | |
| --- | --- | --- | --- | --- | --- | --- | --- | --- | --- | --- | --- | --- | --- | --- | --- | --- | --- |
|  | | | | | | | | **99% Confidence Interval** | | | |  | | | | | |
|  | | **Effect** | | **Estimate** | | **SE** | | **Lower** | | **Upper** | | **df** | | **t** | | **p** | |
|  |  | (Intercept) |  | 0.00 |  | 0.06 |  | -0.16 |  | 0.16 |  | 133.00 |  | 0.00 |  | 1.000 |  |
|  |  | body symptoms - neutral |  | -0.41 |  | 0.07 |  | -0.60 |  | -0.22 |  | 540.00 |  | -5.61 |  | < .001 |  |
|  |  | signs of severe disease - neutral |  | -0.84 |  | 0.07 |  | -1.03 |  | -0.65 |  | 540.00 |  | -11.32 |  | < .001 |  |
|  |  | standard fear - neutral |  | -0.09 |  | 0.07 |  | -0.28 |  | 0.10 |  | 540.00 |  | -1.23 |  | 1.000 |  |
|  |  | bad news - neutral |  | -0.54 |  | 0.07 |  | -0.73 |  | -0.35 |  | 540.00 |  | -7.37 |  | < .001 |  |
|  |  | health anxiety [IAS] |  | 0.16 |  | 0.09 |  | -0.06 |  | 0.39 |  | 133.00 |  | 1.89 |  | 0.310 |  |
|  |  | age |  | 0.05 |  | 0.07 |  | -0.13 |  | 0.22 |  | 133.00 |  | 0.68 |  | 1.000 |  |
|  |  | gender |  | 0.03 |  | 0.07 |  | -0.15 |  | 0.21 |  | 133.00 |  | 0.44 |  | 1.000 |  |
|  |  | anxiety sensitivity [ASI-3] |  | -0.01 |  | 0.10 |  | -0.25 |  | 0.24 |  | 133.00 |  | -0.08 |  | 1.000 |  |
|  |  | somatic symptom severity [PHQ-15] |  | -0.02 |  | 0.09 |  | -0.25 |  | 0.21 |  | 133.00 |  | -0.23 |  | 1.000 |  |
|  |  | depressive symptoms [PHQ-2] |  | -0.12 |  | 0.08 |  | -0.33 |  | 0.10 |  | 133.00 |  | -1.40 |  | 0.815 |  |
|  |  | anxiety symptoms [GAD-2] |  | 0.09 |  | 0.09 |  | -0.15 |  | 0.32 |  | 133.00 |  | 0.92 |  | 1.000 |  |
|  |  | trait anxiety [STAI] |  | 0.01 |  | 0.02 |  | -0.03 |  | 0.05 |  | 133.00 |  | 0.81 |  | 1.000 |  |
|  |  | body symptoms - neutral ✻ health anxiety [IAS] |  | 0.11 |  | 0.10 |  | -0.15 |  | 0.37 |  | 540.00 |  | 1.07 |  | 1.000 |  |
|  |  | signs of severe disease - neutral ✻ health anxiety [IAS] |  | 0.22 |  | 0.10 |  | -0.04 |  | 0.48 |  | 540.00 |  | 2.17 |  | 0.150 |  |
|  |  | standard fear - neutral ✻ health anxiety [IAS] |  | -0.07 |  | 0.10 |  | -0.33 |  | 0.19 |  | 540.00 |  | -0.70 |  | 1.000 |  |
|  |  | bad news - neutral ✻ health anxiety [IAS] |  | 0.18 |  | 0.10 |  | -0.08 |  | 0.44 |  | 540.00 |  | 1.75 |  | 0.400 |  |
|  |  | body symptoms - neutral ✻ anxiety sensitivity [ASI-3] |  | -0.09 |  | 0.11 |  | -0.37 |  | 0.19 |  | 540.00 |  | -0.84 |  | 1.000 |  |
|  |  | signs of severe disease - neutral ✻ anxiety sensitivity [ASI-3] |  | 0.02 |  | 0.11 |  | -0.26 |  | 0.30 |  | 540.00 |  | 0.20 |  | 1.000 |  |
|  |  | standard fear - neutral ✻ anxiety sensitivity [ASI-3] |  | -0.04 |  | 0.11 |  | -0.32 |  | 0.24 |  | 540.00 |  | -0.36 |  | 1.000 |  |
|  |  | bad news - neutral ✻ anxiety sensitivity [ASI-3] |  | -0.13 |  | 0.11 |  | -0.42 |  | 0.15 |  | 540.00 |  | -1.22 |  | 1.000 |  |
|  |  | body symptoms - neutral ✻ somatic symptom severity [PHQ-15] |  | -0.02 |  | 0.10 |  | -0.28 |  | 0.25 |  | 540.00 |  | -0.17 |  | 1.000 |  |
|  |  | signs of severe disease - neutral ✻ somatic symptom severity [PHQ-15] |  | 0.04 |  | 0.10 |  | -0.22 |  | 0.31 |  | 540.00 |  | 0.42 |  | 1.000 |  |
|  |  | standard fear - neutral ✻ somatic symptom severity [PHQ-15] |  | 0.04 |  | 0.10 |  | -0.22 |  | 0.30 |  | 540.00 |  | 0.39 |  | 1.000 |  |
|  |  | bad news - neutral ✻ somatic symptom severity [PHQ-15] |  | 0.17 |  | 0.10 |  | -0.09 |  | 0.44 |  | 540.00 |  | 1.69 |  | 0.460 |  |
|  |  | body symptoms - neutral ✻ depressive symptoms [PHQ-2] |  | -0.02 |  | 0.10 |  | -0.27 |  | 0.22 |  | 540.00 |  | -0.24 |  | 1.000 |  |
|  |  | signs of severe disease - neutral ✻ depressive symptoms [PHQ-2] |  | -0.07 |  | 0.10 |  | -0.31 |  | 0.18 |  | 540.00 |  | -0.71 |  | 1.000 |  |
|  |  | standard fear - neutral ✻ depressive symptoms [PHQ-2] |  | 0.04 |  | 0.10 |  | -0.20 |  | 0.29 |  | 540.00 |  | 0.43 |  | 1.000 |  |
|  |  | bad news - neutral ✻ depressive symptoms [PHQ-2] |  | 0.03 |  | 0.10 |  | -0.22 |  | 0.27 |  | 540.00 |  | 0.27 |  | 1.000 |  |
|  |  | body symptoms - neutral ✻ anxiety symptoms [GAD-2] |  | -0.02 |  | 0.11 |  | -0.30 |  | 0.25 |  | 540.00 |  | -0.22 |  | 1.000 |  |
|  |  | signs of severe disease - neutral ✻ anxiety symptoms [GAD-2] |  | -0.01 |  | 0.11 |  | -0.28 |  | 0.27 |  | 540.00 |  | -0.06 |  | 1.000 |  |
|  |  | standard fear - neutral ✻ anxiety symptoms [GAD-2] |  | -0.07 |  | 0.11 |  | -0.34 |  | 0.20 |  | 540.00 |  | -0.67 |  | 1.000 |  |
|  |  | bad news - neutral ✻ anxiety symptoms [GAD-2] |  | 0.03 |  | 0.11 |  | -0.24 |  | 0.31 |  | 540.00 |  | 0.32 |  | 1.000 |  |
|  |  | body symptoms - neutral ✻ trait anxiety [STAI] |  | 0.03 |  | 0.02 |  | -0.02 |  | 0.07 |  | 540.00 |  | 1.49 |  | 0.680 |  |
|  |  | signs of severe disease - neutral ✻ trait anxiety [STAI] |  | 0.02 |  | 0.02 |  | -0.03 |  | 0.06 |  | 540.00 |  | 0.87 |  | 1.000 |  |
|  |  | standard fear - neutral ✻ trait anxiety [STAI] |  | 0.01 |  | 0.02 |  | -0.03 |  | 0.06 |  | 540.00 |  | 0.81 |  | 1.000 |  |
|  |  | bad news - neutral ✻ trait anxiety [STAI] |  | 0.00 |  | 0.02 |  | -0.04 |  | 0.05 |  | 540.00 |  | 0.13 |  | 1.000 |  |
|  | | | | | | | | | | | | | | | | | |
